# *Pichia pastoris* Aft1 - a novel transcription factor, enhancing recombinant protein secretion

**DOI:** 10.1186/s12934-014-0120-5

**Published:** 2014-09-03

**Authors:** Claudia Ruth, Markus Buchetics, Viktorija Vidimce, Daniela Kotz, Stefan Naschberger, Diethard Mattanovich, Harald Pichler, Brigitte Gasser

**Affiliations:** Austrian Centre of Industrial Biotechnology (ACIB GmbH), Petersgasse 14, 8010 Graz, Austria; Austrian Centre of Industrial Biotechnology (ACIB GmbH), Muthgasse 11, 1190 Wien, Austria; BIOMIN Research Center, Technologiezentrum Tulln, Technopark 1, 3430 Tulln, Austria; Department of Biotechnology, BOKU University of Natural Resources and Life Sciences Vienna, Muthgasse 18, 1190 Vienna, Austria; Institute of Molecular Biotechnology, Graz University of Technology, Petersgasse 14, 8010 Graz, Austria

**Keywords:** *Pichia pastoris*, Aft1, Transcription factor, Novel functions, Enhanced secretion

## Abstract

**Background:**

The methylotrophic yeast *Pichia pastoris* is frequently used for the production of recombinant proteins. However, expression levels can vary depending on the target protein. Allowing for simultaneous regulation of many genes, which may elicit a desired phenotype like increased protein production, overexpression of transcription factors can be used to overcome expression bottlenecks. Here, we present a novel *P. pastoris* transcription factor currently annotated as Aft1, activator of ferrous transport.

**Results:**

The promoter regions of key secretory *P. pastoris* genes were screened for fungal transcription factor binding sites, revealing Aft1 as an interesting candidate for improving secretion. Genome wide analysis of transcription factor binding sites suggested Aft1 to be involved in the regulation of many secretory genes, but also indicated possible novel functions in carbohydrate metabolism. No Aft binding sites were found in promoters of characteristic iron homeostasis genes in *P. pastoris*. Microarrays were used to study the Aft1 regulon in detail, confirming Aft1 involvement in the regulation of carbon-responsive genes, and showing that iron regulation is dependent on *FEP1*, but not *AFT1* expression levels. The positive effect of *AFT1* overexpression on recombinant protein secretion was demonstrated for a carboxylesterase from *Sphingopyxis* sp. MTA144, for which secretion was improved 2.5-fold in fed batch bioreactor cultivations.

**Conclusion:**

This study demonstrates that the transcription factor Aft1 can be used to improve recombinant protein secretion in *P. pastoris*. Furthermore, we discovered possible novel functions of Aft1 in carbohydrate metabolism and provide evidence arguing against a direct role of Aft1 in *P. pastoris* iron regulation.

**Electronic supplementary material:**

The online version of this article (doi:10.1186/s12934-014-0120-5) contains supplementary material, which is available to authorized users.

## Background

The methylotrophic yeast *Pichia pastoris* (syn. *Komagataella phaffii*) is among today’s most frequently used yeast systems for the production of recombinant proteins [1]. Benefits of this yeast are the capability of high cell density cultivations, eukaryotic posttranslational modifications and good secretion capacity. A low level of endogenously secreted proteins allows for the production of relatively pure, recombinant secretory proteins. The recent availability of the genomic sequence boosted the generation of a versatile *P. pastoris* toolbox, including various strains, plasmids and promoters of different strength. To overcome individual bottlenecks during protein folding and secretion, a variety of helper factors such as the ER foldases Pdi1 or BiP (Kar2) have been studied in recent years [2,3]. The capability of transcription factors (TFs) as expression helpers was demonstrated by Guerfal *et al*. and Gasser *et al.* [4,5], who improved the secretion of the mIL-10 protein and antibody fragments by overexpression of the UPR (unfolded protein response) transcription factor *HAC1*. Also overexpression of the gene encoding the TF Nrg1 was shown to positively influence the secretion of recombinant porcine and human trypsinogen as well as the antibody Fab fragment 2 F5 [6].

Allowing for the simultaneous regulation of different proteins involved in e.g. folding and secretion, TFs have huge potential to overcome bottlenecks in the cellular protein production machinery.

Here, we present a novel *P. pastoris* TF which was annotated as Aft1 (Activator of ferrous transport) by sequence homology to *Saccharomyces cerevisiae* Aft1/2. While no information is currently available on the function of *P. pastoris* Aft1, its two *S. cerevisiae* homologs have been studied extensively. In *S. cerevisiae* the transcriptional activators Aft1/2 are responsible for iron uptake and homeostasis fulfilling overlapping but non-redundant roles [7,8]. It was shown that iron homeostasis is primarily maintained by Aft1, while Aft2 is the weaker transcriptional activator [8,9]. Still, both TFs can interact with the same iron-responsive element (FeRE) found within promoters of the genes of the iron regulon such as the iron reductases *FRE1–6* or the multicopper oxidases *FET3/FET5* [8]. Under iron limiting conditions, Aft1 binds to promoters of the iron regulon genes and increases their expression [10,11]. Under iron repletion, the monothiol glutaredoxins (Grx3/4) attached to an iron-sulfur cluster bind Aft1 and initiate its dissociation from target promoters [12]. Export of Aft1 from the nucleus is mediated by the nuclear export receptor Msn5 [13]. Several amino acid (aa) residues have been shown to be important for the iron-responsive regulation of Aft1. While phosphorylation of Ser210/Ser224 and an intermolecular interaction are essential for recognition by Msn5 [13], the residues Leu99, Leu102, Cys291, Cys293 are involved in the interaction with Grx3/4 and iron dependent regulation [12].

In contrast to *S. cerevisiae*, in most fungi such as *Candida albicans*, *Pichia stipitis* or *Schizosaccharomyces pombe*, the iron-regulatory pathway is controlled by a conserved zinc finger GATA-type repressor [14,15]. Also in *P. pastoris* iron uptake was shown to be under control of a GATA-factor, named Fep1. Fep1 was shown to bind to DNA only under iron repletion and disruption of *FEP1* led to constitutively high expression of *FET3*, independent of the availability of iron [16]. Interestingly, several species such as *C. albicans, P. stipitis* and also *P. pastoris* have been found to possess an Aft-type regulator in addition to the GATA-type repressor, leading to the question on the function of Aft in these species [16,17].

In this study, we investigated the functions of the *P. pastoris* Aft1 regulator based on the prediction of putative Aft binding sites in promoters, focusing on the secretion of recombinant proteins. We provide evidence that Aft1 is not directly involved in *P. pastoris* iron regulation, but rather in carbon-responsive regulation. Furthermore, we show that *AFT1*, when overexpressed under its natural promoter, increased the secretion of a model protein up to 2.5-fold in fed batch bioreactor cultivations.

## Results and discussion

### Analysis of transcription factor binding sites in selected genes shown to enhance protein secretion

To identify novel TFs useful for improving protein secretion in *P. pastoris*, the promoter regions, i.e. 1000 bp upstream of the ATG start codon, of key secretory genes involved in folding, transport and exocytosis were studied for putative fungal TF binding sites using the program MatInspector (Genomatix, [18]). The following genes, which were previously identified to improve secretion if modified [4,5,19,20], and/or differentially regulated upon expression of a human sialic acid transporter [21], were studied: *ERO1, HAC1, KAR2, PDI1*, *YDJ1*, *CNE1*, *SSE1*, *SSA4*, *SSB1*, *IRE1*, *UBI4*, *KIN2*, *SSO2*, *CUP5*, *CDS1*, *PGS1*, *ERG1*, *ERG3*, *ERG25*, *NCP1* and *INO1.*

Obtained TF hits were analysed according to frequency, function (link to the secretory machinery or stress response), matrix similarity (similarity of the input sequence to TF matrixes stored in the database, cut-off 0.9) and distance to the start codon (proximal more weighty than distal).

*S. cerevisiae* Aft1/2 binding sites were found enriched in the *P. pastoris* genes *PDI1*, *KAR2*, *SSA4*, *KIN2* and *NCP1* (Table 1). By sequence homology, the gene product of PAS_chr1-4_0361/PP7435_Chr1-1146 [NCBI] was identified as the single *P. pastoris* Aft protein, therefore called Aft1.

**Table 1:** **Putative** ***P. pastoris*** **Aft1 binding sites found in promoters of selected genes known to enhance protein secretion**

**Gene**	**Functions** ^**a**^	**Aft1 UBS (bp)**	**Sequence**
*PDI1*	Protein disulfide isomerase, ER chaperone, formation of disulfide bonds	-423^b^ to -409	gatcacaCACCctct
*KAR2*	ER chaperone, mediating protein folding, UPR regulation	-870^b^ to -856	tcgtataCACCctca
		-194^b^ to -180	tgtaataCACCcttg
*SSA4*	HSP70 protein, co-translational protein-membrane targeting and translocation of nascent proteins into the ER	-608^c^ to -594	actcatgCACCctta
		-342^b^ to -328	catggaaCACCccat
*KIN2*	Serine/threonine kinase involved in exocytosis	-177^c^ to -163	ataactgCACCcaga
*NCP1*	P450 reductase, involved in lipid metabolism (ergosterol biosynthesis)	-539^c^ to -525	ggttttgCACCcagg

^a^Functions are derived for the *S. cerevisiae* homologs of the *P. pastoris* genes in the Saccharomyces Genome Database.

^b^Aft2 matrix.

^c^Aft1 matrix.

Upstream binding sites (UBS): 5′ → 3′, -1000 to -1 A(0)TG. The core binding sequences, representing the highest conserved, consecutive positions are highlighted. TFBS analysis: MatInspector [18] using the search groups fungi and general core promoter elements.

*P. pastoris* Aft1 binding sites were predicted for the regulatory regions of Pdi1 and BiP, two important ER chaperones, indicating that Aft1 is involved in oxidative protein folding. Consistently, Blaiseau *et al.* [7] showed *S. cerevisiae* Aft2 involved in oxidative stress resistance. Also, the chaperone Ssa4 and the serine/threonine kinase Kin2, both recently identified as secretion enhancing factors [3], were found to have an upstream Aft1 binding site, confirming Aft1 as an interesting candidate for improving recombinant protein secretion in *P. pastoris*. In addition, Aft1 binding was predicted for the promoter region of *NCP1*, suggesting that Aft1 is also involved in lipid metabolism [22].

### Prediction of Aft1 binding sites in the *P. pastoris* genome

To elucidate the function of Aft1 in *P. pastoris*, Regulatory Sequence Analysis Tools [23] was used to search for putative Aft1 binding sites in *P. pastoris* promoters. Using the binding motifs known for *S. cerevisiae* Aft1/2, ANTGCACCC and BRCACCCB, resulted in 972 genes with a putative Aft binding site. Thereof, 561 were found annotated [24] and mapped to broader parent terms, GO slim terms, using AmiGO GO Slimmer [25]. Aft1 was found involved in the regulation of approximately 100 different biological processes (Additional file 1), including nucleobase-containing small molecule metabolic processes (57 hits), carbohydrate metabolic processes (50 hits), transcription from RNA polymerase II promoter (47 hits) or amino acids metabolic processes (47 hits). Interestingly, within the categories ion transport (20 hits) and cellular ion homeostasis (11 hits) only 6 proteins with a possible connection to iron regulation were found: Ccc1, mediating transfer of iron from cytosol to vacuole [26]; Gef1, a chloride channel localized to the Golgi or endomembrane system, which has also been reported to be governing iron-limited growth [27]; Hmx1, a heme oxygenase, required for the reutilization of iron from heme, also involved in oxidative stress resistance [28,29] and Nfu1, a protein involved in mitochondrial iron sulfur cluster assembly [30]. Notably, none of these are characteristic iron regulatory genes such as the iron reductases *FRE1-6* or the multicopper oxidases *FET3*/*5*. However, a putative Aft1 binding site was found within the upstream region of *FEP1*, the GATA type repressor, which has been shown to negatively control iron uptake/homeostasis in *P. pastoris* [16]. Thus, though no Aft1 binding site was found within the upstream region of characteristic iron regulators, the presence of such within the upstream region of *FEP1* raises the possibility of a latent, indirect involvement of Aft1 in *P. pastoris* iron regulation.

Analysing possible other functions of *P. pastoris* Aft1, a high number of biological processes related to the cellular secretory pathway machinery was found (Table 2). 23 gene hits were found for protein targeting, 19 for Golgi to vesicle transport, 12 for response to oxidative stress, 11 for folding, 10 for glycosylation and vesicle organization, 9 for vacuole organization and regulation of transport, and 6 for exocytosis. Additionally to the already discussed ER chaperones Pdi1 and BiP, several important secretory regulators were identified. Sec12 and Sec23, both involved in COPII vesicle formation and ER to Golgi transport [31], Sec61, forming a channel for protein translocation into and out of the ER [32], Gos1, a v-SNARE protein found to be involved in ER to Golgi and/or intra-Golgi transport [33], Yap1, a transcription factor involved in oxidative stress response [34], Och1, a Golgi resident mannosyltransferase initiating the hypermannosylation of glycoproteins [35] and Kin1, a protein kinase involved in exocytosis [36], were found to possess a putative upstream Aft1 binding site. In accordance with the above-mentioned data, an Aft1 binding site was found in the upstream region of several genes conferring resistance to oxidative stress, such as *YAP1* or *HMX1*, supporting the hypothesis of a role of Aft1 in oxidative protein folding and/or stress response.

**Table 2:** ***P. pastoris*** **genes with putative Aft1 binding site(s) related to the cellular secretory pathway machinery**

**GO slim term - Biological process**	**Number (Genes)**
GO:0006605 protein targeting	23 (*ATG1 ATG12 ATG18 GET3 GET4 IMP2 KAR2 LHS1 NPL6 NUP188 NUP84 PAM17 PEX14 PEX7 PEX8 SEC61 SPC1 SPC3 TAM41 VPS21 VPS64 VPS68 VPS8*)
GO:0048193 Golgi vesicle transport	19 (*AGE2 APL4 CHS5 DRS2 EMP24 GET3 GOS1 HRR25 KES1 SEC12 SEC23 SFB3 SNC2 SVP26 TED1 TLG1 TRS31 VTI1 YPT32)*
GO:0006979 response to oxidative stress	12 (*AFT1 CTA1 EOS1 GCY1 GSH1 HMX1 HSP104 MXR1 POS5 TSA1 YAP1 YBL055C*)
GO:0006457 protein folding	11 (*CAJ1 CCT2 CCT6 CPR6 EGD2 ERV2 HLJ1 HSP104 PDI1 SIS1 TSA1*)
GO:0006486 protein glycosylation	10 (*ALG7 EOS1 KRE5 MNN11 OCH1 OST6 PMT1 PMT5 STT3 SVP26*)
GO:0016050 vesicle organization	10 (*EMP24 GOS1 SEC12 SEC23 SFB3 SNC2 TLG1 UBC1 VPS4 VTI1*)
GO:0007033 vacuole organization	9 (*ATG1 ATG12 ENO1 GYP7 RDI1 TPM2 VPS21 VTI1 YHC3*)
GO:0051049 regulation of transport	9 (*AKL1 ARG81 CUP9 FPK1 GEF1 SEC12 SEC23 SLG1 TUP1*)
GO:0006887 exocytosis	6 (*ARG81 KES1 KIN1 SNC2 TPM2 YPT32*)

Search field: -1000 bp upstream of the *P. pastoris* GS115 coding sequences. Binding motifs: ScAft1 (ANTGCACCC) and ScAft2 (BRCACCCB). Gene hits were categorized into biological function GO terms using AmiGO GO Slimmer (Yeast GO slim set, [25]).

### *P. pastoris* Aft1 has a conserved Aft-domain, but does not contain the iron-responsive motif

**Figure 1:**
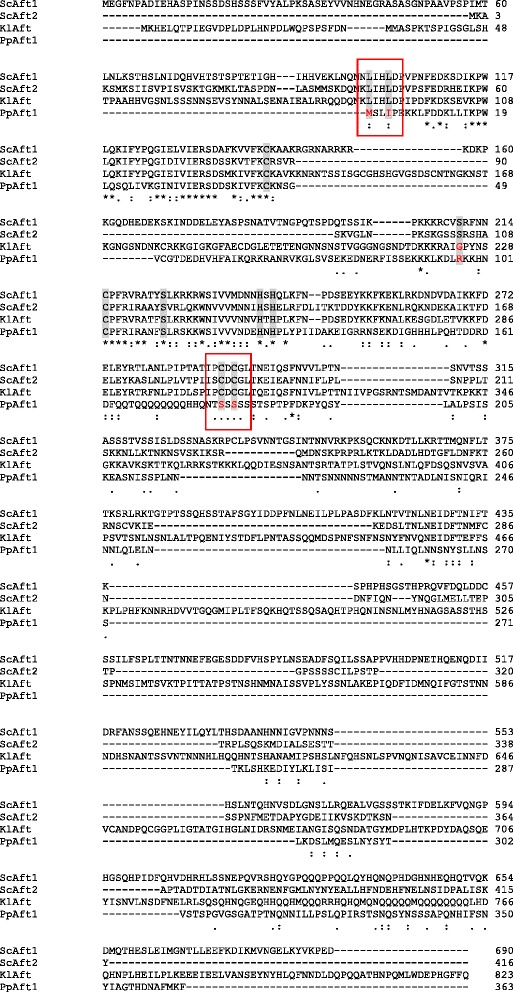
**Aft1 protein sequence comparison.** Amino acid sequences: *S. cerevisiae* Aft1 [NCBI protein: NP_011444] and Aft2 [NCBI protein:NP_015122], *K. lactis* Aft [NCBI protein:CAH00307], and *P. pastoris* Aft1 [NCBI protein:CCA37276] were aligned using ClustalW2 [37]. asterisk: “indicates positions which have a single, fully conserved residue”, colon: “indicates conservation between groups of strongly similar properties”; period: “indicates conservation between groups of weakly similar properties”; shaded grey: conserved residues (Leu99*, Leu102, Cys291 and Cys293 conferring iron sensitivity. Ser210 and Ser224 involved in Msn5 recognition and nuclear export. Cys143, Cys215, His239 and His241, which are suggested to be part of a WRKY-motif involved in zinc binding [39]). Differences are boxed and/or highlighted red; *numbering is based on ScAft1.

The amino acid sequence of *P. pastoris* (Pp) Aft1 was compared to *S. cerevisiae* (Sc) Aft1/2 and *Kluyveromyces lactis* (Kl) Aft using ClustalW2 [37]; all of these proteins display the positive mode of regulation and the characteristic residues Leu99, Leu102, Cys291 and Cys293 conferring iron sensitivity (numbering based on ScAft1, [14]). Interestingly, the *P. pastoris* N-terminal sequence is 41-98 amino acids shorter, lacking the conserved residues Leu99 and Leu102 (PpAft1: Met1, Ile4; Figure 1). Also the residues Cys291 and Cys293 were not found conserved in the *P. pastoris* sequence (PpAft1: Ser180, Ser182), suggesting a role different from iron regulation for PpAft1. Still, also regions of high homology were found in the N-terminal part of the *P. pastoris* protein, particularly the Aft-domain (supposedly the DNA binding domain [38]) between the amino acids 10-130. Especially high conservation was found for the residues 10-45 (identity 53%) and 102-130 (identity 45%), including the two conserved cysteines and histidines, which have been suggested to be part of a WRKY-motif involved in zinc binding [14,39]. Regarding nuclear export, no conservation was found for Ser210 in *P. pastoris* (PpAft1: Arg97) and *K. lactis* (KlAft: Gly224), but Ser224 is conserved in all 4 sequences (PpAft1: Ser111, KlAft: Ser238). However, as Ueta *et al.* [13] showed that individual serine mutations did not affect ScAft1 localization, export via Msn5p seems still possible for PpAft1 and KlAft. In contrast to ScAft1 and KlAft, a glutamine-rich region was found not at the C-terminal end, but between the residues 164-177. This glutamine-rich region is followed by serine (aa 180-213) and asparagine repeats (aa 216-269), maybe as part of a protein-protein interaction domain [40]. No homology was found for the C-terminal part of the PpAft1 protein (identity <1% ), in particular the residues 162 to 363. Interestingly, with only 363 amino acids PpAft1 is considerably shorter than ScAft1 (690 aa), ScAft2 (416 aa) and KlAft (823 aa).

PpAft1 was further compared to *C. albicans* Aft [NCBI protein: XP_714862], which also lacks the characteristic residues Leu99, Leu102, Cys291 and Cys293. Similar to *P. pastori*s, in *C. albicans* iron regulation was shown to be under control of a GATA-type repressor [17]. Sequence comparison of these two proteins revealed only one short N-terminal region of high homology between the residues 3-47 (identity 67%, data not shown). This region was also found to be highly similar in ScAft1/2 and KlAft. Additionally, we searched the genome of the genetically and biochemically close methylotrophic yeast *Hansenula polymorpha* for a homolog of PpAft1. The protein encoded by HPODL_04658 in *H. polymorpha* (NCBI protein: ESX01890) has high N-terminal sequence homology including the two conserved cysteines und histidines (aa 3-132: identity 55%, Additional file 2). A region of high similarity was also found between the residues 244-332 (identity 31%), suggesting that HPODL_04658 functions similar to PpAft1. As PpAft1 and CaAft, also HPODL_04658 lacks the characteristic iron sensitivity residues. Accordingly, we also found a homolog of the GATA-type repressor in the *H. polymorpha* genome (HPODL_03720; 48% sequence identity to PpFep1). In summary, Aft homologs from species that are known to contain also a Fep1-like GATA type repressor (*C. albicans*, *Pichia stipitis*, *Debaryomyces hansenii* according to [14] and *H. polymorpha*) share high sequence homology to PpAft1 in the N-terminal DNA binding region, but lack the characteristic residues that are conferring iron sensitvity in ScAft1/2 (data not shown). Interestingly, all these species possess another protein of unknown function having a domain with sequence similarity to parts of the Aft DNA-binding domain, but low similarity to ScAft1/2 (i.e. less than 15% overall identity).

### The *P. pastoris* Aft1 regulon

**Figure 2:**
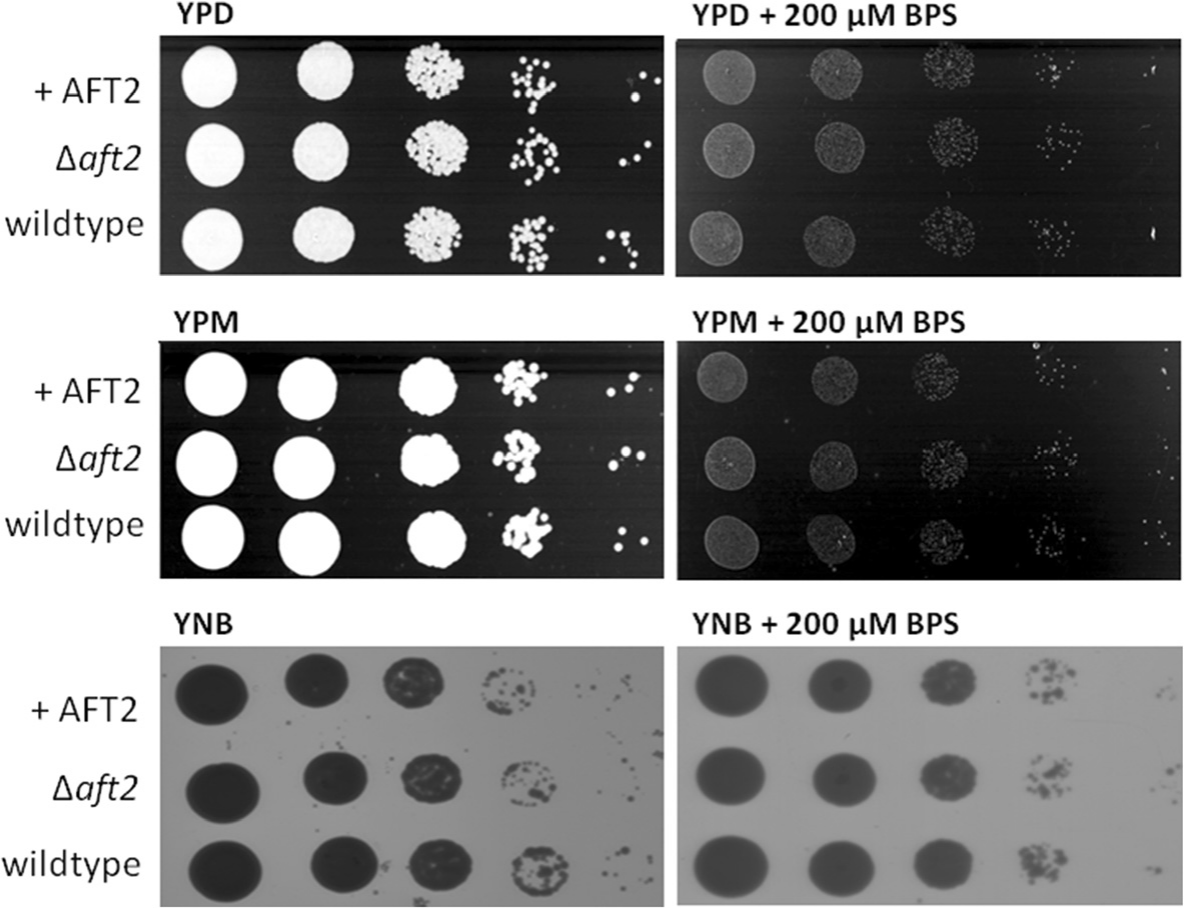
**Spotting assay on iron deficient media.** Serial 1:10 dilutions of *P. pastoris* CBS7435 wild type, AFT1-OE and Δ*aft1* cell suspensions were spotted on YP plates containing either 2% glucose or 1% methanol as carbon source and on YNB agar plates containing 2% glucose as carbon source. Iron-deficient conditions were generated by addition of the iron chelator BPS (200 μM). Plates were incubated at 28°C for 70 h. While addition of BPS resulted in generally delayed growth on both substrates, no difference between the strains was seen.

To investigate if *P. pastoris* Aft1 is involved in iron regulation, we generated an *AFT1* overexpression (AFT1-OE) and an Δ*aft1* disruption mutant, and tested their growth in low and high iron containing media. AFT1-OE was achieved by expressing an additional copy of *AFT1* under control of its native promoter (see below), while the Δ*aft1* strain was generated by exchanging parts of the gene for the KanMX marker cassette using the split marker approach as described by Heiss *et al.* [41]. Low iron media was prepared by addition of the iron chelator BPS (bathophenanthroline disulphonate) to YPD or YNB-Glucose agar plates as described by Miele *et al.* [16]. In order to exclude the possibility to be outside the sensitivity range we tried several concentrations of BPS in the iron sensitivity assay. Miele et al. [16] reported the use of YPD containing 80–160 μM BPS for *P. pastoris*. We tested concentrations ranging from 80-200 μM BPS in YP and minimal medium (YNB or M2) using either glucose or methanol as carbon source. We did not observe growth impairment of *P. pastoris* CBS7435 wild type using 80 μM BPS in YPD or YPM (data not shown). When using higher BPS concentrations, growth of all strains was significantly delayed. However, we did not observe differences between the Δ*aft1* strain, the AFT1-OE and the wild type control on any of the media tested (Figure 2). Thus, disruption of *AFT1* does not render *P. pastoris* sensitive to iron-limited conditions. This behaviour is contrary to the phenotype observed for Δ*aft1/2* in *S. cerevisiae* and Δ*aft1* in *K. lactis*, which grow only poorly or are unable to grow in the same low iron conditions [7,14], suggesting that *P. pastoris* Aft1 is not involved in iron regulation. Additionally, the *P. pastoris* Δ*aft1* mutant did not show impaired growth on the cell wall disturbing agent Calcofluor White, which is again in contrast to what is reported for *S. cerevisiae* Δ*aft1* (not shown).

In order to identify the regulon of Aft1 in *P. pastoris*, we analysed the transcription patterns of the Δ*aft1* strain and control using DNA microarrays. All strains were cultivated in synthetic minimal medium with glucose feed beads for 5 h in three biological replicates. Interestingly, although Aft-like proteins are described as transcriptional activators, an even higher number of genes was up-regulated (54 genes, 33 thereof more than 1.5-fold) than down-regulated (34 genes, 13 thereof more than 1.5-fold) in the Δ*aft1* mutant compared to the wild type (an adjusted p-value of < 0.05 was applied as cut-off to identify significantly regulated genes, Additional file 3). 42% of the up-regulated and 38% of the down-regulated genes contain at least one putative Aft1 binding site in their promoters (compared to 18% of total *P. pastoris* genes), suggesting that both, up- and down-regulation, are a direct consequence of *AFT1* disruption. Table 3 summarizes differentially regulated genes in the Δ*aft1* mutant compared to the wild type strain according to their GO term category.

**Table 3:** **Differentially expressed and annotated genes in the Δ** ***aft1*** **vs. control (wild type) strain**

**Up-regulated genes (Δ** ***aft1*** **vs. control)**	
**GO slim term - Biological process**	**Number (Genes)**
GO:0006811 ion transport	7 ( *AQR1 ATX1 GEF1 MEP2 TPO3 YHL008C ZRT1* )
GO:0008150 biological_process*	6 ( *ECM13 GPM3 YEL023C YLR156C-A YLR278C YOR292C* )
GO:0055085 transmembrane transport	6 ( *AQR1 GEF1 MEP2 STL1 TPO3 ZRT1* )
GO:0006366 transcription from RNA polymerase II promoter	5 ( *GCR1 PHD1 RPM2 UGA3 YLR278C* )
GO:0042221 response to chemical	5 ( *AQR1 ATX1 GCR1 SLI1 UGA3* )
GO:0006520 cellular amino acid metabolic process	4 ( *ADH2 ECM4 SFA1 UGA3* )
GO:0007124 pseudohyphal growth	4 ( *HMS1 MEP2 PHD1 PTP1* )
GO:0051186 cofactor metabolic process	4 ( *ADH2 ALD4 BIO2 THI21* )
GO:0005975 carbohydrate metabolic process	3 ( *DOG1 GCR1 GPM3* )
GO:0006091 generation of precursor metabolites and energy	2 ( *GCR1 GPM3* )
GO:0006766 vitamin metabolic process	2 ( *BIO2 THI21* )
GO:0006873 cellular ion homeostasis	2 ( *ATX1 GEF1* )
GO:0055086 nucleobase-containing small molecule metabolic process	2 ( *ADH2 ALD4* )
GO:0001403 invasive growth in response to glucose limitation	1 ( *PTP1* )
GO:0002181 cytoplasmic translation	1 ( *RPM2* )
GO:0006325 chromatin organization	1 ( *GCR1* )
GO:0006397 mRNA processing	1 ( *RPM2* )
GO:0006470 protein dephosphorylation	1 ( *PTP1* )
GO:0006629 lipid metabolic process	1 ( *YJU3* )
GO:0006865 amino acid transport	1 ( *AQR*1 )
GO:0006979 response to oxidative stress	1 ( *ATX1* )
GO:0007005 mitochondrion organization	1 ( *RPM2* )
GO:0008033 tRNA processing	1 ( *RPM2* )
GO:0008643 carbohydrate transport	1 ( *STL1* )
GO:0070647 protein modification by small protein conjugation or removal	1 ( *PCI8* )
**Down-regulated genes (Δ** ***aft1*** **vs. control)**	
**GO slim term - Biological process**	**Number (Genes)**
GO:0006811 ion transport	4 ( *ATO2 JEN1 TAT2 VMA8* )
GO:0055085 transmembrane transport	4 ( *ITR1 JEN1 PEX13 TAT2* )
GO:0006281 DNA repair	3 ( *PCD1 RVB1 YRA1* )
GO:0006325 chromatin organization	3 ( *ACS1 RLF2 RVB1* )
GO:0006366 transcription from RNA polymerase II promoter	3 ( *MIG1 RVB1 YHP1* )
GO:0006974 cellular response to DNA damage stimulus	3 ( *PCD1 RVB1 YRA1* )
GO:0008150 biological_process*	3 ( *NBA1 TOS8 YHR177W* )
GO:0005975 carbohydrate metabolic process	2 ( *CTS1 MIG1* )
GO:0006766 vitamin metabolic process	2 ( *SNO1 SNZ3* )
GO:0051186 cofactor metabolic process	2 ( *ACS1 SNZ3* )
GO:0055086 nucleobase-containing small molecule metabolic process	2 ( *PCD1 YRA1* )
GO:0070271 protein complex biogenesis	2 ( *PEX13 RLF2* )
GO:0000278 mitotic cell cycle	1 ( *YHP1* )
GO:0000910 cytokinesis	1 ( *CTS1* )
GO:0002181 cytoplasmic translation	1 ( *RPL4A* )
GO:0006091 generation of precursor metabolites and energy	1 ( *ACS1* )
GO:0006520 cellular amino acid metabolic process	1 ( *SNO1* )
GO:0006605 protein targeting	1 ( *PEX13* )
GO:0006865 amino acid transport	1 ( *TAT2* )
GO:0006873 cellular ion homeostasis	1 ( *VMA8* )
GO:0007031 peroxisome organization	1 ( *PEX13* )
GO:0008643 carbohydrate transport	1 ( JEN1 )
GO:0015931 nucleobase-containing compound transport	1 ( *YRA1* )
GO:0016570 histone modification	1 ( *ACS1* )
GO:0018193 peptidyl-amino acid modification	1 ( *ACS1* )
GO:0042221 response to chemical	1 ( *MIG1* )
GO:0042594 response to starvation	1 ( *MIG1* )
GO:0043543 protein acylation	1 ( *ACS1* )
GO:0048193 Golgi vesicle transport	1 ( *BRE5* )
GO:0051049 regulation of transport	1 ( *BRE5* )
GO:0051169 nuclear transport	1 ( *YRA1* )
GO:0070647 protein modification by small protein conjugation or removal	1 ( *BRE5* )

*Biological process is unkown.

Genes were categorized into biological function GO terms using AmiGO GO Slimmer (Yeast GO slim set, [25]).

In the Δ*aft1* strain, 9 putative transcriptional regulators are among the regulated genes, seven of them carrying putative Aft1 binding sites in their promoters. PAS_chr4_0324, a fungal specific transcription factor of unknown function with a Zn2/Cys6 DNA-binding domain is the highest up-regulated gene, while *MIG1-1*, encoding a transcription factor connected to glucose repression, has lower expression levels in the Δ*aft1* mutant. Most other regulated transcription factors are also of the fungal-specific Zn2/Cys6 type, but lack annotated function and defined target genes. Furthermore, 23 of the 54 up-regulated and 9 of the 34 down-regulated genes encode hypothetical proteins of unknown function. The second largest group of regulated genes comprises transport proteins, mainly transmembrane transporters, however, there is no clear preference for transported substrates, which range from ions to polyamines and sugars.

Regarding iron regulation, no changes in transcript levels were observed for the GATA-type repressor *FEP1*, in line with the unaltered expression levels of genes involved in iron uptake and homeostasis (i.e. *FRE1–6* or *FET3/FET5*). Re-analysis of our previous microarray data (obtained in different environmental conditions, [42,43]) for expression changes of iron regulatory genes indeed revealed that induction of the iron regulon is dependent on the levels of *FEP1*, but not *AFT1* in *P. pastoris*.

Notably, Atx1 and Gef1, which were described to be required for the correct assembly of the high affinity iron transporter Fet3 in *S. cerevisiae*, were found among the up-regulated genes in the Δ*aft1* mutant, pointing towards a different regulation of iron acquisition genes in *P. pastoris*.

Among the up-regulated genes several genes which are described to be repressed by glucose in *S. cerevisiae* are found, including alcohol dehydrogenase *ADH2* and the two putative mitochondrial aldehyde dehydrogenases *ALD4-1* and *ALD5*. All of these contain putative Aft1 binding sites in their promoter regions. Interestingly, deletion of these gene functions has recently been predicted by the genome scale metabolic model of *P. pastoris* to enhance recombinant protein production [44]. Moreover, carbon-source responsive transporters of the multifacilitator superfamily are among the regulated genes in the Δ*aft1* mutant. While the genes encoding the high affinity glucose transporter Ght1 and the putative glycerol transporter PAS_c034_0021 are up-regulated in the Δ*aft1* mutant, the second high affinity glucose transporter of *P. pastoris*, encoded by *GHT2* as well as a homolog of the *S. cerevisiae* myo-inositol transporter *ITR1*, are repressed in this strain. This implicates that Aft1 is involved in the regulation of glucose-repressed genes, at least in an indirect manner by regulating *MIG1-1* expression, but maybe also directly as some of these genes also contain Aft1 binding sites in their promoters. In this respect, it should be noted that *AFT1* expression levels are significantly higher not only in glucose-limited conditions, but also in cells grown on methanol as compared to glucose or glycerol surplus (see below and own unpublished data). A correlation of *AFT1/2* expression levels and the carbon source has also been observed in *S. cerevisiae*, where differences between the fermentable carbon source glucose and the non-fermentable carbon source glycerol were reported [38].

### Regulation of the *P. pastoris* AFT1 promoter

To study the expression strength of the natural *AFT1* promoter and to test if it is applicable for overexpression studies, intracellular expression of green fluorescent protein was analysed (cycle-3-GFP, [45]). 96-well deep-well plate screening, which has been applied for *P. pastoris* promoter studies before [46], was used for cultivation. However, the protocol was adjusted for *GAP* promoter driven expression, which was used for the expression of our model protein carboxylesterase.

**Figure 3:**
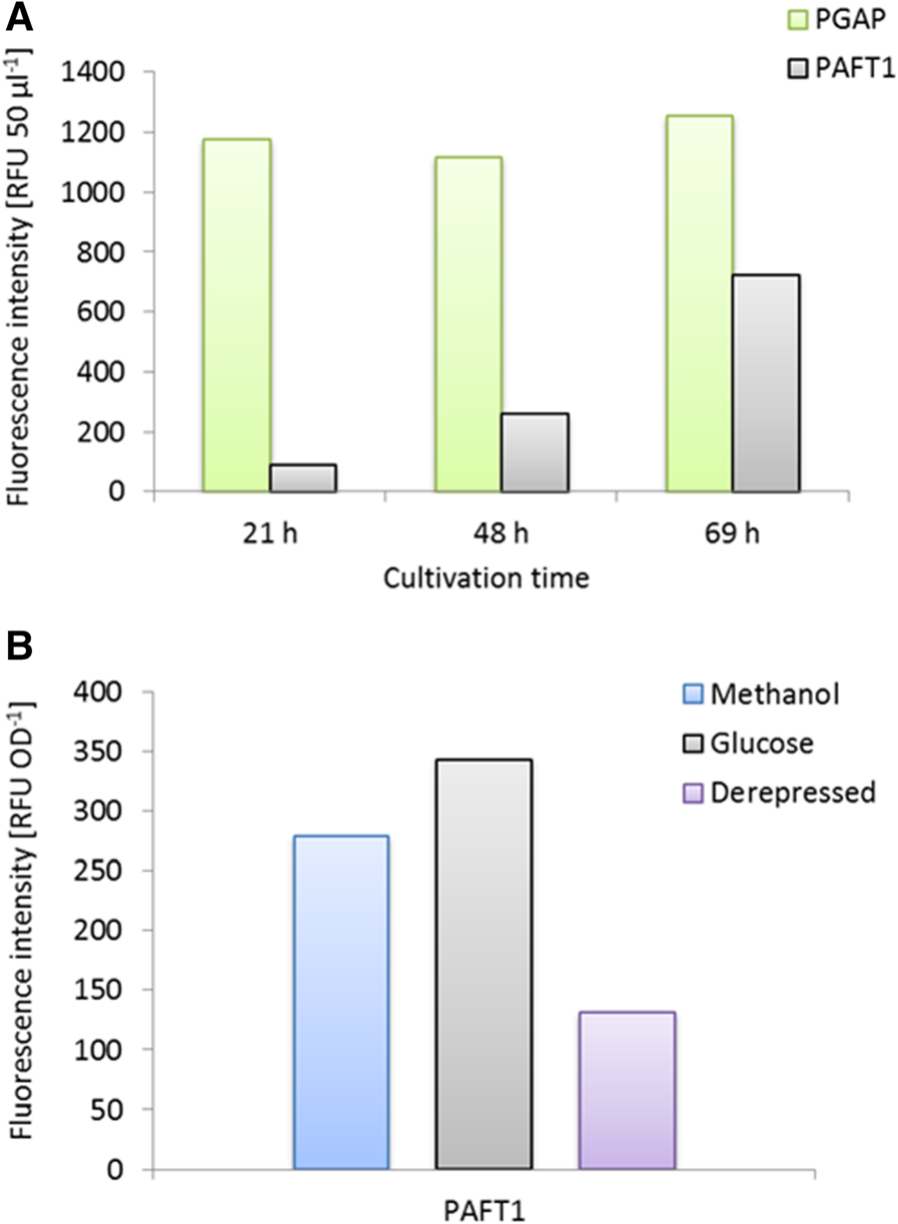
**Characterization of the** ***AFT1*** **promoter. (A)** GFP expression levels under control of P_*GAP*_ and P_*AFT1*_. Cultivation in 96-well plate. RFU: relative fluorescence units, average of 87 *P. pastoris* clones. **(B)** GFP expression levels under control of P_*AFT1*_ using different cultivation conditions. Glucose: cultivation time 69 h, 2% glucose batch, 3 times addition of glucose to 0.5%. Methanol: cultivation time 92 h, 48 h glucose batch (1%), 3 times addition of methanol to 0.5%. Derepressed: cultivation time 69 h, 2% glucose batch, no additional supplementation.

*P. pastoris* was transformed with plasmid pPpKan-S-GFP. Clones expressing GFP from the *AFT1* promoter were studied for intracellular fluorescence levels in comparison to clones expressing GFP from the constitutive *GAP* promoter. On average, the tested P_*AFT1*_ clones reached 724 relative fluorescence units (RFU) after 69 h of cultivation, which was 60% in comparison to the respective P_*GAP*_ clones. In terms of average yields, the P_*AFT1*_ clones reached 343 RFU OD^-1^, corresponding to 50% of the yield of the P_*GAP*_ clones, characterising P_*AFT1*_ as fairly strong promoter under the conditions tested. Analysing expression at 24, 48 and 69 h of cultivation, differences in regulation were observed. While the P_*GAP*_ clones showed the expected constitutive expression profile with stable titers of 1201 ± 64 RFU at the different time points, P_*AFT1*_ controlled expression significantly increased with longer cultivation times, reaching a maximum at 69 h of cultivation (Figure 3A). Assuming that the observed effect was related to a decrease in glucose concentration, a batch series starting with 1, 2 and 4% glucose was studied for about 48 h (data not shown). The results confirmed enhanced P_*AFT1*_ activity at lower glucose concentrations, showing average yields of 104, 81 and only 16 RFU OD^-1^, for the 1, 2 and 4% glucose batches, respectively.

Interestingly and matching above data, the *AFT1* promoter was also found inducible by methanol, reaching 80% (280 RFU OD^-1^) of the yield of glucose cultivated cells (Figure 3B). In comparison, using a 2% glucose batch without supplementation (derepressed protocol), a yield of only 40% was reached. While P_*AFT1*_ is obviously a rather strong and also methanol-inducible promoter, unlimited access of glucose negatively affects P_*AFT1*_ activity and presumably decreases overexpression effects.

### The effect of *AFT1* overexpression on the secretion of recombinant proteins

Although none of the secretion-related genes was differentially expressed in the Δ*aft1* mutant under the analysed conditions, our next step was to analyse whether *AFT1* overexpression influences the secretion of recombinant proteins. Our model protein of choice was a carboxylesterase from *Sphingopyxis* sp. MTA144. This carboxylesterase is under development for use as a feed additive enzyme, because it hydrolyses an antinutritive substance that may be naturally contained in animal feed [47]. *P. pastoris* has recently been shown to secrete active carboxylesterase [48]. However, for a technological application of this carboxylesterase as feed enzyme for gastrointestinal detoxification in animals, a high yield recombinant production process is required.

### Generation of carboxylesterase secreting strains

Carboxylesterase secreting strains were generated by transformation of *P. pastoris* CBS7435 with plasmid pPM2dZ30-PGAPα-CE expressing carboxylesterase under control of the *GAP* promoter and using the *S. cerevisiae* alpha mating factor signal sequence as secretion leader. Carboxylesterase represented the major fraction of total secreted protein of each transformant, yielding a strong band at the calculated size of 52 kDa. However, due to varying numbers of integrated expression cassettes, strains secreting lower and higher levels of carboxylesterase were observed (Additional file 4). The best-secreting strain CE#18 contained 6 copies of the expression cassette, while the average strain CE#12 had only one expression cassette integrated in its genome, confirming a positive correlation between secretion level and gene copy number. Using ELISA, an expression level of 80 μg mL^-1^ was determined for CE#18, whereas a titer of approximately 20 μg mL^-1^ was determined for strain CE#12.

### *AFT1* co-expression studies

**Figure 4:**
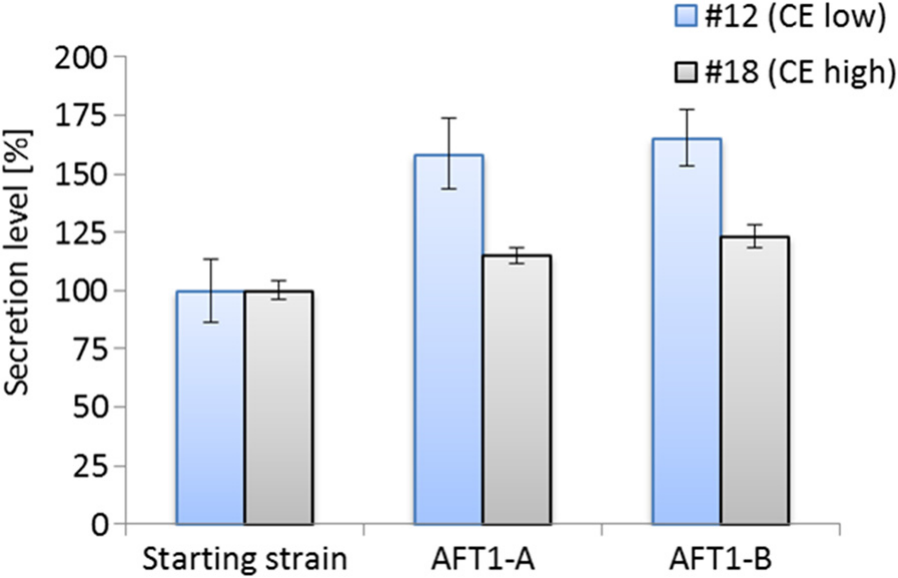
**Screening results of improved carboxylesterase-AFT1** ***P. pastoris*** **strains.** Secretion levels: determined by ELISA and normalized to the respective starting strain. Starting strains: CE#12 (blue, low level expressing strain), CE#18 (grey, high level expressing strain). AFT1-A and AFT1-B: *AFT1* overexpressing clones.

The strains CE#12 and CE#18 were chosen to study whether overexpression of *AFT1* can positively influence recombinant protein secretion. Both strains were transformed with linearized pPM2aK21-AFT1, comprising the *AFT1* gene under control of its natural promoter and terminator. Supernatants of 8 transformants of each strain were studied for carboxylesterase secretion after 48 h of cultivation in shake flask. ELISA was used to reveal small, but significant differences in carboxylesterase secretion levels, showing improved secreting clones for both strain backgrounds. Figure 4 shows the results of the best two clones of each strain. Interestingly, while the best CE#12-AFT1 clones yielded a strong improvement of 62 ± 5%, the best two CE#18-AFT1 strains showed considerably less improvement, reaching an increase of only 19 ± 6%. However, it has to be considered that strain CE#18 produced four times more carboxylesterase (80 μg mL^-1^) than strain CE#12 (20 μg mL^-1^). Assuming that Aft1 is involved in oxidative protein folding, high overproduction of carboxylesterase could have led to an overload of recombinant protein in the ER and subsequent induction of ERAD. Newly elicited upstream or downstream bottlenecks could also be the reason for the reduced influence of *AFT1* overexpression and the only modest improvement for strain CE#18.

The growth behaviour of *AFT1* overexpressing strains was studied in addition. In contrast to secretion levels, growth under these conditions was seemingly unaffected by overexpression of *AFT1*, showing neither significant changes in final OD levels (<15% , 48 h) nor any detectable alteration in the growth curve (data not shown).

### Bioreactor cultivations

To confirm the positive effect of *AFT1* overexpression on carboxylesterase secretion, the best strains from screening were studied in bioreactor cultivations. First, the performance of the *AFT1* overexpressing strain CE#12-AFT1-A was compared to the starting strain (CE#12). Cultivations were performed using aerobic and hypoxic production conditions as preceding bioreactor experiments had indicated that a low oxygen supply is beneficial for carboxylesterase secretion (data not shown), an observation that had also been made for the production of antibody Fab fragments and trypsinogen [49]. While under aerobic production conditions a dissolved oxygen (DO) concentration of 20% was maintained throughout the whole process, a DO level of 5% was applied during the feed phase for hypoxic conditions. Bioreactor cultivations were monitored for 100 h. In addition to the quantification of secreted recombinant carboxylesterase by SDS-PAGE, also the functional quality of the enzyme was assessed by an activity assay.

**Figure 5:**
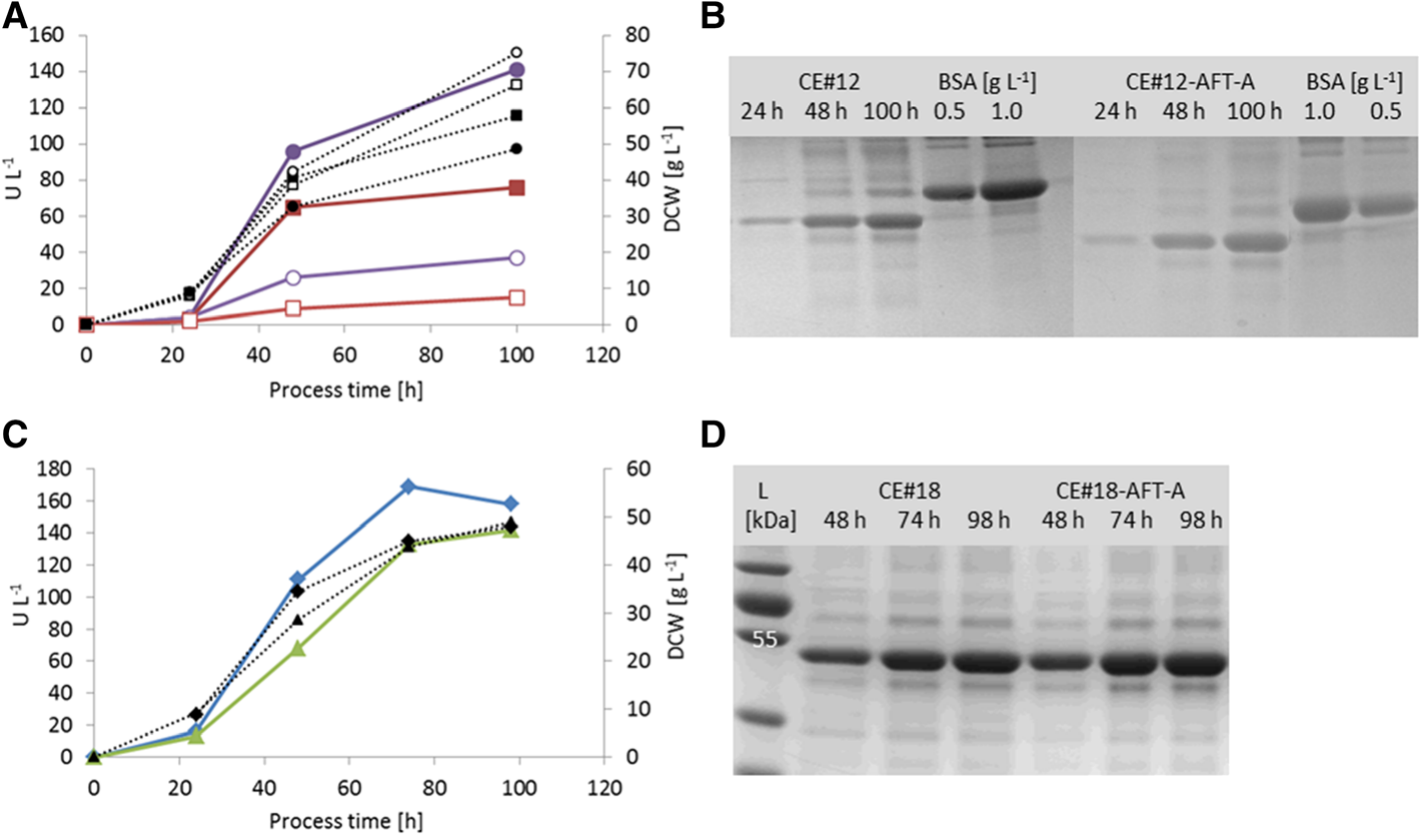
**Bioreactor cultivations of improved carboxylesterase-AFT1** ***P. pastoris*** **strains. (A)** Carboxylesterase activity levels (U L^-1^) of strain CE#12 (red squares) and strain CE#12-AFT1-A (purple circles). Closed symbols: hypoxic conditions; open symbols: aerobic conditions. **(C)** Carboxylesterase activity levels (U L^-1^) of strain CE#18 (green triangles) and strain CE#18-AFT1-A (blue rhomboids). Dashed lines: biomass values (DCW, g L^-1^). **(B)** and **(D)** Reducing SDS-PAGE of supernatant samples from different cultivation time points (hypoxic conditions). BSA: bovine serum albumin standard (66.5 kDa).

The positive effect of *AFT1* overexpression on the secretion rate of strain CE#12 was indeed confirmed in bioreactor. A maximum of 141 U L^-1^ was reached for strain CE#12-AFT1-A after 100 h of cultivation under hypoxic conditions, while the starting strain reached only a level of 76 U L^-1^ at this time point (Figure 5A). Confirming the beneficial effect of hypoxic production conditions, carboxylesterase activity levels were 3-4 fold lower under aerobic conditions. Applying 20% DO, a maximum of only 37 U L^-1^ was reached for strain CE#12-AFT1-A after 100 h of cultivation. However, comparing to the starting strain under the same conditions a 2.5-fold higher activity level was achieved for the *AFT1* overexpressing strain. It seems that the amount of dissolved oxygen does not abrogate the beneficial impact of *AFT1* overexpression on carboxylesterase secretion. As observed previously [43], lower amounts of biomass were reached in hypoxic compared to normoxic conditions due to the production of ethanol at the low DO setpoint. Consistently, about 2-fold higher carboxylesterase yields (U g^-1^ biomass) were obtained for the *AFT1* overexpressing strain under both conditions. The results of the activity assays were confirmed by SDS-PAGE (Figure 5B). Comparing to a bovine serum albumin (BSA) standard, a carboxylesterase protein level of about 0.75 g L^-1^ was estimated for strain CE#12-AFT1-A after 100 h of cultivation under hypoxic conditions, while the starting strain produced below 0.5 g L^-1^ until this time point.

Also the performance of the multi-copy carboxylesterase *AFT1* overexpressing strain CE#18-AFT1-A was investigated in bioreactor under hypoxic conditions (Figure 5C). Again the positive effect of *AFT1* overexpression was confirmed. Strain CE#18-AFT1-A reached 169 U L^-1^ after 74 h of cultivation, while the starting strain (CE#18) only reached 133 U L^-1^ at the same time point. As before, results were confirmed by SDS-PAGE, showing increased protein levels for strain CE#18-AFT1-A after 74 and 98 h of cultivation (Figure 5D). Comparing yields, secretion per biomass was increased 24% for strain CE#18-AFT1-A. The *AFT1* overexpressing strain also yielded 25% higher ethanol levels, reconfirming the involvement of *P. pastoris* Aft1 in carbon-responsive regulation.

The *AFT1* copy number of the overexpressing strains was also investigated. RT-PCR revealed one *AFT1* overexpression cassette for strain CE#12-AFT1-A and three for strain CE#18-AFT1-A. Notably, strain CE#18-AFT1-B, which had only one *AFT1* overexpression cassette integrated, did not show superior behaviour in bioreactor (data not shown). Seemingly, several *AFT1* expression cassettes are necessary to positively influence the secretion of the high-level carboxylesterase producer #18, which might allow for further improvement by targeted *AFT1* copy number amplification or the use of stronger promoters for *AFT1* overexpression.

Both carboxylesterase-strains, CE#12 and CE#18, were also transformed with an empty vector control plasmid (pPM2aK21-empty). However, carboxylesterase secretion levels were not influenced, neither in shake flask (CE#12-empty) nor in bioreactor cultivations (CE#18-empty).

## Conclusions

By analysing the promoter regions of secretion enhancing *P. pastoris* genes, the ortholog of the *S. cerevisiae* iron regulators Aft1/2 was selected as a novel factor to improve recombinant protein secretion. A genome-wide analysis of putative Aft1 binding sites in *P. pastoris* showed Aft1 to be involved in the regulation of many secretory genes, in addition to genes involved in carbohydrate metabolism. The absence of Aft1 binding sites in iron regulatory genes, i.e. *FRE1–6* or *FET3/FET5*, led us to assume iron-independent functions of Aft1 in *P. pastoris*. These findings were supported by primary amino acid sequence analysis, showing that the DNA binding domain, but not the iron-responsive motif is conserved in the *P. pastoris* protein. Using DNA microarrays we unveiled further evidence that Aft1 might not directly be required for iron regulation, but is rather involved in regulatory mechanisms in response to carbon source availability, showing e.g. higher transcriptional activation at low glucose concentrations. We also discovered an involvement of *P. pastoris* Aft1 in the expression of glucose-repressible genes, which needs to be analysed in more detail in future studies. Finally, though microarray data did not reveal differential regulation of any secretion related genes, the secretion enhancing effect of *AFT1* was confirmed in overexpressing strains, yielding up to 2.5-fold more secreted carboxylesterase.

## Materials and methods

### Strains and plasmids

*P. pastoris* wild type CBS7435 (Centraalbureau voor Schimmelcultures, NL) was used as host strain [50]. The plasmid pPpKan-S-GFP for GFP expression was described by Näätsaari *et al.* ([GenBank:JQ519694], [51]). Plasmid pPM2dZ30-PGAPα, a derivative of pPUZZLE [6], was used for the expression of a carboxylesterase from *Sphingopyxis macrogoltabida* (aa residues 48 to 540, [GenBank: ACS27056]) under control of the *P. pastoris GAP* promoter, with the *S. cerevisiae* α-MF leader sequence for secretion and a Zeocin resistance marker cassette. Prior to transformation, the expression vector was linearized within the *GAP* promoter using the restriction enzyme *Bln*I for homologous integration into the native *GAP* promoter locus of the *P. pastoris* genome.

For overexpression of *P. pastoris AFT1* the pPUZZLE derived plasmid pPM2aK21 was used, which contains the KanMX4 cassette conferring resistance to Kanamycin/Geneticin (G418), and an *AOX1* terminator sequence, which, when linearized with *Asc*I, provides the homologous stretches for integration into the native *P. pastoris AOX1* terminator locus. The *AFT1* expression cassette, including the *AFT1* gene [*P. pastoris* gene identifier:PAS_chr1-4_0361] and 1000 bp up- and 400 bp downstream sequences, was amplified from *P. pastoris* genomic DNA using following primers: ApaI-AFT-fw (AAAGGGCCCCCAGGTGAATGTACGTAATGGAG) and AgeI-AFT-rv (TTTACCGGTGGGGAGAAGCCGAATTGGAAG). After *Apa*I/*Age*I digestion, the PCR product was cloned into pPM2aK21, creating pPM2aK21-AFT.

### *AFT1* gene knock out

A split marker cassette approach was used as described by Heiss *et al.* [41] to generate transformants with a disrupted *AFT1* gene locus. The *AFT* flanking regions (A upstream, D downstream) were fused to the G418 resistance cassette fragments B and C, respectively, by overlap PCR. Then, equal amounts of both split marker fragments (AB and CD) were pooled and simultaneously transformed into *P. pastoris*. The G418 resistance gene is reconstituted when both split marker fragments integrate at the correct locus. Verification of positive Δ*aft1* transformant strains was done by PCR using a primer pair designed to bind on the native *AFT1* locus up- and downstream of the split-marker cassette on genomic DNA of Geneticin-resistant transformants. Primers and split marker fragment sizes are shown in Additional file 5.

### Transformation

Electrocompetent *P. pastoris* cells were transformed using the following parameters: 1.5-2.0 kV, 25 μF and 200 Ω. After two hours of regeneration on YP medium, containing 20 g L^-1^ glucose, selection of positive transformants was done by incubation for 48 h and 28°C on YPD agar plates (20 g L^-1^ yeast extract, 10 g L^-1^ peptone, 20 g L^-1^ glucose, 20 g L^-1^ agar-agar) supplemented with antibiotic, 50 μg mL^-1^ Zeocin and/or 450 μg mL^-1^ Geneticin, respectively.

### Media and cultivation

If not stated otherwise chemicals were purchased from BD, Carl Roth, Merck and Sigma Aldrich. YP medium contained 20 g peptone and 10 g yeast extract per liter. Buffered minimal (BM)-medium contained 10 g yeast extract, 10 g peptone, 100 mM potassium phosphate buffer (pH 6.0), 13.4 g yeast nitrogen base without amino acids and 0.4 mg biotin per liter.

Microscale cultivations for GFP expression were performed in 96-well deep-well plates. 300 μL of BM-medium, containing 2% glucose, were inoculated using a toothpick and incubated at 25°C and 360 rpm. The culture was supplemented with glucose to 0.5% at 24 and 36 h of cultivation. Cells were finally harvested after 69 h of cultivation. Methanol induced cultivations were carried out based on the protocol of Weis *et al.* [52]. Cells were grown for 48 h in 300 μL of BM-medium, containing 1% glucose. Cells were induced by addition of methanol (0.5%) after 48, 56 and 72 h of cultivation. Finally, cells were harvested after 92 h of cultivation.

Shake flask cultivations were performed in 100 mL shake flasks without baffles. As pre-culture, 2.5 mL YP medium, containing 2% glycerol and the respective antibiotic(s), were inoculated in a 50 mL falcon tube and incubated for a minimum of 24 h at 25°C and 180 rpm. The main cultures, containing 10 mL BM-medium with 2% glucose, were inoculated to an OD_600_ of 0.1. Cultures were then incubated at 25°C and 180 rpm, and supplemented three times with glucose to 0.5% in 12 h intervals. Cells were harvested after 48 h by centrifugation at 4000 rpm. Subsequently, supernatants were analyzed for carboxylesterase production and cell pellets were used for cell weight analysis. Alternatively, OD_600_ was measured.

Pre-cultures for bioreactor cultivations were performed in 500 mL baffled shake flasks and 50 mL YP medium, containing 2% glucose and 0.01 g L^-1^ Glanapon DG160 (Bussetti). The main medium used for fed batch cultivations was used as described by Zhao *et al.* [53], supplemented with biotin and Glanapon DG160. The batch medium consisted of 4 g L^-1^ KH_2_PO_4_, 4 g L^-1^ (NH_4_)_2_SO_4_, 0.38 g L^-1^ CaCl_2_, 18.2 g L^-1^ K_2_SO_4_, 9.4 g L^-1^ MgSO_4_^.^7H_2_O, 40 g L^-1^ glucose monohydrate (Tereos Syral), 1 g L^-1^ Glanapon DG160 and 1 mL L^-1^ trace element solution. The trace element solution contained 2.50 g L^-1^ MnSO_4_^.^H_2_O (Riedel-de-Haën), 54.17 g L^-1^ FeSO_4_^.^7H_2_O (Merck), 16.67 gL^-1^ ZnCl_2_^.^2H_2_O (Riedel-de-Haën) and 0.17 gL^-1^ Na_2_MoO_4_^.^2H_2_O. The batch medium was supplemented with 2 mL of 0.2 g L^-1^ biotin stock solution per liter medium. The feed medium consisted of 600 g L^-1^ glucose monohydrate, 2 g L^-1^ (NH_4_)_2_PO_4_ and 1 g L^-1^ Glanapon DG160 and was supplemented with 2 mL of biotin stock solution per liter medium.

High cell-density fermentations were carried out in 1 L bioreactors (DASGIP). A starting volume of 500 mL batch medium was inoculated to an OD_600_ of 0.3. The pH was measured using a glass electrode (Mettler Toledo) and maintained at 5.0 ± 0.1 by automatic addition of 25% ammonium hydroxide (AppliChem). Dissolved oxygen (DO) was monitored using an optical, dissolved oxygen electrode (Hamilton) and maintained at 20% of saturation during the batch phase by a DO cascade of agitation and aeration. Hypoxic conditions were applied during the feed phase. Maximum agitation was 1200 rpm (corresponding to a maximum tip speed of 2.89 m s^-1^); maximum aeration was set to 1.3 vvm. Temperature was maintained at 25°C.

### SDS-PAGE and western blot

10 μL of culture supernatant (containing secreted carboxylesterase enzyme) were run on a reducing sodium dodecyl sulfate (SDS) NuPAGE® 12% Bis-Tris polyacrylamide gel (Life technologies™) with NuPAGE® morpholinepropanesulfonic acid (MOPS) buffer at 180 V for 60 min. Protein bands were visualized using Coomassie staining solution. For Western blotting, SDS-PAGE separated proteins were transferred to a nitrocellulose membrane using the XCell II™ Blot Module for wet (tank) transfer (Life technologies™) according to the manufacturer’s instructions. Carboxylesterase was detected using anti-carboxylesterase antiserum as described by Heinl *et al.* [48].

### Gene copy number determination using real time PCR

Copy numbers were determined by Real time (RT)-PCR as described by Abad *et al.* [54]. The ABI PRISM 7300 Real Time PCR System and Power SYBR® Green PCR Master Mix were used (Life technologies™). Normalization of the data was achieved using the *P. pastoris ARG4* gene as reference. The number of copies per μL was calculated using Avogadro’s number. Following primers were used for amplification: ARG4-RTfw (TCCTCCGGTGGCAGTTCTT), ARG4-RTrv (TCCATTGACTCCCGTTTGAG), AFT-RTfw (GGGCAATATCCAATAGGGCTAA), AFT-RTrv (GGGTGCGCCAAGACTAACA), ZEO-RTfw (CGGCCTGGACGAGCTGTA), ZEO-RTrv (GGCTGCTCGCCGATCTC). Genomic DNA was prepared according to Hoffman and Winston [55].

### Fluorescence measurements

GFP fluorescence levels were determined per 50 μL of cell culture. Measurements were performed in microplate using the SynergyMX plate reader (Biotek) applying the following settings: excitation 395 nm and emission 507 nm.

### Carboxylesterase activity assay

Enzymatic activity was determined photometrically at 405 nm by hydrolysis of p-Nitrophenyl 2-(trimethylsilyl) ethyl carbonate (pNSi) to p-Nitrophenol + 2-(trimethylsilyl) ethanol + carbon dioxide. The reaction was performed at 37°C in microtiter plates and followed over time using a Tecan Sunrise™ plate reader (XFLUOR4 version: V 4.51). Clarified fermentation supernatants were used undiluted and in several dilutions (10^-1^ - 10^-5^) using 1 × FCE buffer. 10 × FCE buffer consisted of 200 mM Tris-Cl, pH 8.0 and 1 mg mL^-1^ BSA. 100 mM pNSi stock solution was prepared by dissolving 141.68 mg pNSi in 5 mL of 96% ethanol. 1 mM pNSi reaction solution was made by mixing 100 mM pNSi stock solution with 1× FCE buffer. 20 μL of the test samples were provided in a microtiter plate and the reaction was started by addition of 180 μL reaction solution. Absorbance was measured every 30 s for 2 h and activity was determined according to Lambert-Beer’s law.

### Iron-dependent growth analysis: spotting assay

The ferrous iron chelator Bathophenanthrolinedisulfonate disodium salt (BPS; Sigma) reduces the amount of free iron in the medium. YP agar plates containing 80 - 200 μM BPS were prepared by addition of BPS to the melted YP or YPD (YP + 2% glucose) agar right before pouring the plates. Methanol (1%) was applied directly in 250 μL volumes onto YP agar plates and spread using a sterile spatula. YNB agar plates containing 200 μM BPS were prepared by addition of BPS to the melted YNB agar right before pouring the plates. YNB agar contained 3.4 g L^-1^ Yeast Nitrogen Base (Becton Dickinson, NJ), 10 g L^-1^ ammonium sulfate, 20 g L^-1^ glucose and 100 mM potassium phosphate buffer (pH 6.0). Cells from a YPD agar plate were resuspended in 1 mL sterile PBS, the optical density (OD_600_) was determined and set to OD_600_ = 0.3. Five μL of serial 1:10 dilutions (in sterile PBS) were applied on each agar plate. The plates were then incubated at 30°C for 72 h.

### Regulatory sequence analysis tools (RSAT)

RSAT subcategory pattern matching and string genome-scale dna-pattern was used to search for the *S. cerevisiae* Aft1 and Aft2 binding sites within -1000 bp upstream of the *P. pastoris* GS115 coding sequences. Default settings were applied.

### AmiGO Go Slimmer

AmiGO Go Slimmer (version 1.8) was used to map genes into GO slim terms according to their biological process. SGD was used as database filter (Evidence Code: all) and Yeast GO slim as pre-existing GO slim set (GO database release: 27.10.2012 (analysis of *P. pastoris* Aft1 binding sites) and 15.07.2013 (analysis of microarray data: *Δaft1* vs. control). Advanced results option was used to display gene products and counts for each slim term.

### Microarray hybridization and data analysis

For the generation of samples for microarray analysis the Δ*aft1* strain and the wild type control were cultivated in three biological replicates. Pre-cultures were cultivated as described above. The main cultures, containing 2 mL M2 medium, were inoculated to an optical density OD_600_ of 4.0 and a 12 mm glucose FeedBead (Kuhner Shaker) was added. The synthetic medium M2 contained per liter: 22.0 g Citric acid monohydrate, 3.15 g (NH_4_)_2_PO_4_, 0.49 g MgSO_4_*7H2O, 0.80 g KCl, 0.0268 g CaCl_2_*2H_2_O, 1.47 mL of PTM1 trace metals and 4 mg Biotin; pH was set to 5 with KOH (solid). Cultures were shaken at 180 rpm and 25°C. Slow release of glucose ensured glucose limited growth. Samples were taken after five h of main culture (estimated specific growth rate: 0.08 h^-1^), fixed in phenol/ethanol (5% phenol (v/v) in pure ethanol, ice-cold), and stored at -80°C until total RNA extraction.

Total RNA extraction was performed using Trizol as described in Graf *et al.* [42]. cDNA synthesis and labelling as well as the microarray hybridizations (in-house designed *P. pastoris* specific oligonucleotide arrays, AMAD-ID 034821, 8x15K custom arrays, Agilent) were carried out according to the Agilent protocols Quick Amp Labelling Kit (Cat. No. 5190-0444) and Gene Expression Hybridisation Kit (Cat. No. 5188-5242) using a reference design. Therefore, each sample was labelled in a dye-swap manner and hybridized against a reference cDNA, which was generated from a pool of cells grown under different culture conditions. Normalization steps and statistical analysis of microarray data included removal of color bias using locally weighted MA-scatterplot smoothing (LOESS) followed by between array normalization using the “Aquantile” method. For identifying differentially expressed genes and calculating p-values a linear model fit with an eBayes correction was used. P-values were adjusted for multiple testing with the false discovery method (FDR) by Benjamini & Yekutieli. For identifying differentially expressed genes, a fold change cut-off of at least 1.5 > FC >1/1.5 was applied. All steps were done using the R software (http://www.rproject.org) and the limma package. The expression changes of some genes selected based on their regulation pattern was confirmed using quantitative real time PCR (Additional file 6).

## Additional files

Additional file 1:
***P. pastoris*** **genes with putative Aft1 binding site(s).** Search field: -1000 bp upstream of the *P. pastoris* GS115 coding sequences. Binding motifs: ScAft1 (ANTGCACCC) and ScAft2 (BRCACCCB). Genes were categorized into biological function GO-Terms using AmiGO GO Slimmer (Yeast GO slim set, [25]). Genes not found in the database and removed from the calculation: *FLO104*, *ATG30*, *SHL23* and *FEP1*. Additional file 2:
**Aft1 protein sequence comparison (ClustalW2, [** 37 **]).** Amino acid sequences: *P. pastoris* Aft1 [NCBI protein:CCA37276] and *H. polymorpha* HPODL_04658 [NCBI protein: ESX01890]; asterisk: “indicates positions which have a single, fully conserved residue”, colon: “indicates conservation between groups of strongly similar properties”; period: “indicates conservation between groups of weakly similar properties”; red boxed: conserved residues (Cys45*, Cys102, His126 and His128), shaded grey: Aft domain (pfam_ls:AFT, Motif Scan-My Hits-SIB), *numbering is based on PpAft1. Additional file 3
**List of significantly regulated genes in Δ** ***aft1*** **compared to wild type.** GO term enrichment was analysed using Amigo GO Slimmer [25]. Significantly regulated GO terms were determined by GO term Finder. List of homologs of the *S. cerevisiae* iron regulon which are not regulated in Δ*aft1* in *P. pastoris*. Additional file 4:
**SDS-PAGE of carboxylesterase secreting** ***P. pastoris*** **strains.** Clones #8-20: CBS7435 transformed with plasmid pPM2dZ30-PGAP-CE; negative control: CBS7435. Additional file 5:
**Primers used for the construction of the split marker cassette for** ***AFT1*** **disruption in** ***P. pastoris.***
Additional file 6:
**Primers used for the verification of gene regulation in Δ** ***aft1*** **and wild type.** Comparison of gene regulation patterns from microarrays and qPCR for genes selected based on their regulatory behaviour.
