# Supplementary material for: Pichia pastoris Aft1 - a novel transcription factor, enhancing recombinant protein secretion
Source: Microb Cell Fact. 2014 Sep 3;13:120. doi: 10.1186/s12934-014-0120-5 (PMC4161868; doi:10.1186/s12934-014-0120-5)
Supplement: Additional file 1: — P. pastoris genes with putative Aft1 binding site(s). Search field: -1000 bp upstream of the P. pastoris GS115 coding sequences. Binding motifs: ScAft1 (ANTGCACCC) and ScAft2 (BRCACCCB). Genes were categorized into biological function GO-Terms using AmiGO GO Slimmer (Yeast GO slim set, [25]). Genes not found in the database and removed from the calculation: FLO104, ATG30, SHL23 and FEP1. [file 12934_2014_120_MOESM1_ESM.pdf]

GO Slim Term - Biological Process	Number (Genes)
GO:0055086 nucleobase-containing small molecule metabolic process	57 (*ADE12 ADE17 ADH2 ADK1 AGE2 ALD4 ATP1 ATP2 CAB4 CDC21 DAL2 DDP1 DYN1 FAD1 FDH1 GET3 GNA1 GND2 GPD1 GTS1 GUF1 GYP1 GYP7 LHS1 MDH3 MSB4 NDE2 NMA1 NNT1 PCC1 PCD1 PHO8 PNC1 POS5 PRS4 PXA1 PYC2 QRI1 RAS1 RIA1 SAM2 SEC12 SEC23 SER1 SNQ2 SOL1 SQS1 SUA5 TKL1 TPI1 TUB1 TUB2 URA3 XPT1 YKL151C YNK1 YPK9*)
GO:0005975 carbohydrate metabolic process	50 (*ALG13 ALG7 BGL2 CHS5 CIT1 DCW1 DDP1 DGA1 DOG1 ENO1 EOS1 FBA1 FBP26 GCY1 GLC7 GLK1 GND2 GPD1 GUT1 HSP104 ICL1 INM2 KRE5 KRE6 MDH1 MDH3 MLS1 MNN11 NDE2 OCH1 OST6 PFK2 PKP1 PMT1 PMT5 PPG1 PYC2 RBK1 ROT2 SCW4 SOL1 SOR1 SPR1 STT3 SVP26 TKL1 TPI1 UTR2 XKS1 YBR056W*)
GO:0008150 biological_process	48 (*ACO2 AIM45 CRP1 EHD3 FMP41 GDT1 HRQ1 HSP31 NIT1 NIT2 OCA2 PBY1 PCD1 RTC1 SEF1 TIR3 TMA17 TMT1 UBX3 YBR056W YCR051W YDL144C YDL206W YDR262W YDR336W YDR541C YER156C YET1 YJR107W YJR111C YLR241W YLR253W YLR278C YLR419W YMR034C YMR221C YNL115C YOL098C YOR093C YOR292C YPK9 YPL109C YPL162C YPL247C YPR114W AOX1 AOX2*)
GO:0006366 transcription from RNA polymerase II promoter	47 (*AFT1 ARG81 ARO80 BUR6 CCR4 CUP9 ELP4 EMI5 GLC7 GTS1 HAP2 HAP5 INO80 ITC1 KAR4 NOT3 NPL6 NUP84 PCC1 RFX1 RLM1 ROX1 RPD3 RPT1 RSC9 RTR1 RTT109 RVB1 SEF1 SEN1 SFP1 SGV1 SIN4 SPT16 SPT4 SPT5 SRB7 STP2 SWD2 TAF5 THO2 THP1 TUP1 UGA3 YAP1 YHP1 YLR278C*)
GO:0006520 cellular amino acid metabolic process	47 (*AAT1 AAT2 ABZ1 ACO1 ADH2 ADI1 ALD2 ARG81 ARO1 ARO7 ARO80 ARO9 BAT1 CAR1 CIT1 CPA2 ECM4 EHD3 GAP1 GCV2 GLN1 GSH1 HIS2 HOM3 HYP2 IDH1 IDH2 ILV1 ISM1 KRS1 MAE1 MES1 MET10 MET17 MET5 PET112 PLB3 SAH1 SAM2 SER1 SER3 STR3 TRP1 TRP3 UGA3 YHR020W ZRC1*)
GO:0051186 cofactor metabolic process	40 (*ABZ1 ACH1 ACO1 ACO2 ACS2 ADH2 ALD4 CAB4 CIT1 COQ5 EMI5 FAA1 FAD1 FAU1 FDH1 GND2 GPD1 HEM14 HEM2 HMX1 ICL1 IDH1 IDH2 KGD2 MDH1 MDH3 MLS1 NDE2 NMA1 NNT1 PHO8 PNC1 POS5 PYC2 SAM2 SDH1 SOL1 TKL1 TPI1 YKL151C*)
GO:0023052 signaling	39 (*ACK1 AGE2 BCK1 BUD3 DPP1 FRQ1 GET3 GTS1 GYP1 GYP7 MSB4 NPR3 PKP1 PTC2 PTP3 RAS1 RDI1 RGD1 RHO2 RHO4 RLM1 SAP190 SLG1 SRV2 SSK22 STE7 STT4 TAP42 TEM1 TFS1 TIP41 TPK2 TRL1 TUS1 VPS21 VPS34 VPS64 YPT10 YPT32*)
GO:0006091 generation of precursor metabolites and energy	37 (*ACO1 ACO2 ADH2 AIM45 ALG7 CIT1 COQ10 COQ5 COR1 COX5B CYB2 CYC1 CYT1 EMI5 ENO1 ETR1 FBA1 GLC7 GLK1 HAP2 HAP5 ICL1 IDH1 IDH2 KGD2 MAM33 MDH1 MDH3 MET10 MLS1 NDE2 PET100 PFK2 PPG1 PUF3 SDH1 TPI1*)
GO:0042221 response to chemical stimulus	37 (*AFT1 BCK1 CTA1 EOS1 FPK1 GCY1 GET3 GSH1 HAP2 HAP5 HSP104 ITC1 KAR2 KAR4 LHS1 MXR1 PCC1 PMT1 POS5 PRM1 PTC2 RGD1 SNQ2 SSY1 STE23 STE24 STE7 TRL1 TSA1 TUB2 TUP1 UGA3 VPS64 YAP1 YBL055C YCK2 ZRC1*)
GO:0055085 transmembrane transport	37 (*AGC1 ANT1 ATP1 ATP2 ESBP6 GAP1 GEF1 HOL1 HXT6 ITR2 JEN1 KAR2 LEU5 LHS1 MCD4 MIR1 NHA1 NHX1 ODC1 PAM17 PIC2 PUT4 PXA1 SEC61 SSY1 STL1 SUL1 TAM41 TAT2 THI73 TPO3 TPO4 VNX1 YDL119C YDL206W YPR011C ZRC1*)
GO:0006629 lipid metabolic process	36 (*ALG13 ANT1 ARE2 AYR1 CKI1 DAP1 DGA1 DGK1 DPP1 ERG13 ERG2 ERG27 ERG5 ETR1 FAA1 FAS1 FOX2 GTS1 IFA38 INM2 KES1 MCD4 MDH3 NCP1 NTE1 PLB3 SPT14 STT4 TAM41 TGL3 TGL4 TSC10 TUP1 VPS34 VPS4 YJR107W*)
GO:0006974 response to DNA damage stimulus	35 (*APN1 CDC7 CHK1 GLC7 HRR25 HTA2 INO80 MCM2 MCM4 NPL6 NUP84 PLM2 PSF2 PTC2 RAD10 RAD26 RAD5 RFA1 RFA2 RFC4 RPL40A RPS31 RTT109 RVB1 SGS1 SIR2 SPT16 SPT4 SPT5*

	THO2 THP1 TPP1 TSA1 TUP1 YNK1)
GO:0006364 rRNA processing	31 (*DHR2 EFG1 EMG1 ESF2 GLC7 LSM3 MAK16 NOC3 NOP1 NOP14 POP1 POP7 REX2 RLP7 RPP1 RPS18A RPS1B RPS2 RPS31 RPS6B RRP40 RRP5 RRS1 RSC9 SEN1 SNU13 SPT4 SPT5 SQS1 UTP15 UTP6*)
GO:0000278 mitotic cell cycle	30 (*BFR1 CCR4 CDC10 CDC16 CDC7 CLB4 DBF4 DYN1 EFG1 GLC7 HRR25 IRR1 KAR4 MCM2 MCM4 PCL2 RPD3 RSC9 SAP190 SDA1 SGS1 SSD1 SWE1 TEM1 TRF5 TUB1 TUB2 YBP2 YHP1 ZPR1*)
GO:0007005 mitochondrion organization	30 (*ACO1 ARP3 COA3 ILM1 IMG1 IMG2 IMP2 ISM1 KGD2 MDM1 MRP10 MRP2 MRPL19 MRPL3 MRPL35 MRPS18 MSS51 NAT3 OXA1 PAM17 PET100 PET112 PET191 PUF3 REX2 RIM1 RSM23 TAM41 TPM2 YME2*)
GO:0006468 protein phosphorylation	29 (*AKL1 ATG1 BCK1 CDC11 CDC7 CHK1 CLB4 DBF4 FPK1 HRR25 IME2 KCC4 KIN1 KIN3 NCE102 PCL2 PKP1 PTC2 PTP3 RIM11 SGV1 SSK22 STE7 SWE1 TPK2 VPS34 YAK1 YCK2 YCK3*)
GO:0006281 DNA repair	28 (*APN1 CDC7 HRR25 HTA2 INO80 MCM2 MCM4 NUP84 PSF2 RAD10 RAD26 RAD5 RFA1 RFA2 RFC4 RPL40A RPS31 RTT109 RVB1 SGS1 SIR2 SPT16 SPT4 SPT5 THO2 THP1 TPP1 YNK1*)
GO:0070271 protein complex biogenesis	27 (*ARP3 CDC10 COA3 CRN1 KCC4 MRP10 MSS51 NAS2 NCE102 OST6 OXA1 PET100 PET191 PEX14 PEX7 PRE2 RAD5 RAV1 RPN2 RPN6 RPT1 SFB3 SPT16 TUB1 TUB2 TWF1 VPS4*)
GO:0071554 cell wall organization or biogenesis	26 (*ACK1 BGL2 CHS5 DCW1 ECM14 ECM21 ECM4 IRR1 KRE5 KRE6 MCD4 MET5 PRS4 RHO2 ROT2 RRT12 SCW4 SLG1 SPR1 SSD1 STE7 SVP26 TUS1 UTH1 UTR2 YPS1*)
GO:0045333 cellular respiration	25 (*ACO1 ACO2 ALG7 CIT1 COQ10 COQ5 COR1 COX5B CYC1 CYT1 EMI5 ETR1 HAP2 HAP5 ICL1 IDH1 IDH2 KGD2 MAM33 MDH1 MDH3 MLS1 PET100 PUF3 SDH1*)
GO:0006397 mRNA processing	24 (*BUD13 CBC2 CCR4 GLC7 LSM3 MPE1 MSS51 NAB2 NOT3 POP1 POP7 PRP21 RPP1 RSE1 SEN1 SNU13 SPT4 SPT5 SQS1 SWD2 THO2 THP1 TRL1 YME2*)
GO:0007010 cytoskeleton organization	24 (*AIP1 AKL1 ARP3 CDC10 CLB4 CRN1 DYN1 HRR25 KCC4 MSB4 NAT3 PCL2 PIN3 RDI1 RHO2 RHO4 RVS161 SDA1 SLG1 SRV2 SWE1 TPM2 TWF1 YSC84*)
GO:0006605 protein targeting	23 (*ATG1 ATG12 ATG18 GET3 GET4 IMP2 KAR2 LHS1 NPL6 NUP188 NUP84 PAM17 PEX14 PEX7 PEX8 SEC61 SPC1 SPC3 TAM41 VPS21 VPS64 VPS68 VPS8*)
GO:0006260 DNA replication	22 (*ADK1 CCR4 CDC16 CDC7 CHK1 DBF4 GLC7 MCM2 MCM4 NOC3 ORC1 ORC2 PRI1 PSF2 RFA1 RFA2 RFC4 RIM1 RPD3 SGS1 SIR2 SPT16*)
GO:0006325 chromatin organization	22 (*ACS2 ARD1 GLC7 HTA2 HTB2 INO80 ITC1 NPL6 ORC2 PAA1 RPD3 RSC9 RTT109 RVB1 SGV1 SIR2 SPT16 SPT4 SWD2 TAF5 TUP1 UTH1*)
GO:0006811 ion transport	20 (*AGC1 ANT1 ATP1 ATP2 CCC1 DRS2 GEF1 HOL1 JEN1 MIR1 NHA1 NHX1 PFK2 PIC2 SUL1 VNX1 YDL206W YHL008C YPK9 ZRC1*)
GO:0051603 proteolysis involved in cellular protein catabolic process	20 (*CDC16 EPS1 HLJ1 KAR2 MCA1 NAS2 PMT1 PRE1 PRE2 PRE3 RPN2 RPN6 RPN7 RPT1 SEC61 SSM4 UBC1 UBC4 UBP16 YUH1*)
GO:0048193 Golgi vesicle transport	19 (*AGE2 APL4 CHS5 DRS2 EMP24 GET3 GOS1 HRR25 KES1 SEC12 SEC23 SFB3 SNC2 SVP26 TED1 TLG1 TRS31 VTI1 YPT32*)
GO:0051321 meiotic cell cycle	18 (*BFR1 CDC7 DBF4 EMI5 GLC7 HRR25 IME2 KAR4 MSC1 NPR3 RAD10 RFA1 RFA2 RPD3 SGS1 SWE1 TUB1 TUB2*)
GO:0051726 regulation of cell cycle	18 (*BFR1 CCR4 CDC7 CHK1 CLB4 DBF4 GLC7 IME2 KCC4 PCL2 PTC2 RPD3 SDA1 SGS1 SWE1 TEM1 TSA1 ZPR1*)

GO:0042274 ribosomal small subunit biogenesis	17 (*DHR2 DRS2 EFG1 EMG1 ESF2 HRR25 NOP14 RPS18A RPS1B RPS31 RPS6B RRP5 RRS1 SNU13 SQS1 UTP15 UTP6*)
GO:0002181 cytoplasmic translation	16 (*RBG2 RPL11A RPL16A RPL1A RPL26A RPL2A RPL40A RPL4A RPL5 RPL9B RPS18A RPS1B RPS25A RPS31 RPS6B TMA46*)
GO:0033043 regulation of organelle organization	16 (*ARP3 BFR1 CLB4 COA3 CRN1 ENO1 GLC7 GYP7 MSS51 RDI1 RHO4 SEC12 SEC23 SGV1 SWE1 TWF1*)
GO:0048285 organelle fission	16 *(BFR1 CDC16 CDC7 CLB4 DBF4 DYN1 GLC7 HRR25 IRR1 SGS1 SSD1 SWE1 TEM1 TRF5 TUB1 TUB2*)
GO:0008033 tRNA processing	15 (*CCA1 DUS3 ELP4 HRR25 KTI11 LOS1 LSM3 NOP1 POP1 POP7 RPP1 SAP190 SEN1 SUA5 TRL1*)
GO:0006417 regulation of translation	14 (*COA3 GCD1 GCD6 GCN1 GUF1 HYP2 MSS51 RPS2 SSD1 SUA5 SUI1 TRL1 TSA1 YGR054W*)
GO:0006897 endocytosis	13 (*AKL1 DRS2 ECM21 GTS1 KES1 RVS161 SLG1 SNC2 TLG1 UBC1 VPS21 YCK2 YSC84*)
GO:0015931 nucleobase-containing compound transport	13 (*ANT1 BUD13 CBC2 LOS1 MCD4 NAB2 NUP188 NUP84 RPS18A RPS2 SOL1 THO2 THP1*)
GO:0018193 peptidyl-amino acid modification	13 (*ACS2 ARD1 CPR6 HYP2 KTI11 NAT3 NMT1 OST6 PKP1 RRT2 RTT109 SFM1 TAF5*)
GO:0032543 mitochondrial translation	13 (*COA3 IMG1 IMG2 ISM1 MRP10 MRP2 MRPL19 MRPL3 MRPL35 MRPS18 MSS51 PET112 RSM23*)
GO:0051169 nuclear transport	13 (*BUD13 LOS1 NAB2 NPL6 NUP188 NUP84 RPS18A RPS2 RRS1 SDA1 SOL1 THO2 THP1*)
GO:0000746 conjugation	12 (*CHS5 GET3 IRR1 KAR2 KAR4 PRM1 RVS161 SCW4 STE7 TUB1 TUB2 VPS64*)
GO:0006310 DNA recombination	12 (*CDC7 MCM2 MCM4 MSC1 PSF2 RAD10 RFA1 RFA2 RPD3 SGS1 SIR2 THO2*)
GO:0006979 response to oxidative stress	12 (*AFT1 CTA1 EOS1 GCY1 GSH1 HMX1 HSP104 MXR1 POS5 TSA1 YAP1 YBL055C*)
GO:0008380 RNA splicing	12 (*BUD13 CBC2 LOS1 LSM3 MSS51 PRP21 RSE1 SNU13 SPT4 SPT5 SQS1 TRL1*)
GO:0043934 sporulation	12 (*CDC10 CHS5 EMI5 GLC7 IME2 PRB1 PTP3 RIM11 RRT12 SEF1 SPR1 TGL4*)
GO:0000910 cytokinesis	11 (*BUD13 BUD3 CDC10 CDC11 CHS5 KCC4 RAX2 THP1 TWF1 UTH1 YCK2*)
GO:0006457 protein folding	11 (*CAJ1 CCT2 CCT6 CPR6 EGD2 ERV2 HLJ1 HSP104 PDI1 SIS1 TSA1*)
GO:0006873 cellular ion homeostasis	11 (*AFT1 CCC1 GEF1 GLC7 HMX1 NFU1 NHA1 NHX1 RAV1 YHC3 ZRC1 FEP1*)
GO:0007059 chromosome segregation	11 (*DBF4 DYN1 GLC7 HRR25 IRR1 KIN3 RFC4 SGS1 SPT4 TUB1 TUB2*)
GO:0006354 DNA-dependent transcription, elongation	10 (*CCR4 NOT3 NPL6 RPD3 RSC9 SPT16 SPT4 SPT5 THO2 THP1*)
GO:0006401 RNA catabolic process	10 (*CBC2 CCR4 LSM3 MRPL3 NOT3 PUF3 RRP40 SKI3 THP1 TRF5*)
GO:0006486 protein glycosylation	10 (*ALG7 EOS1 KRE5 MNN11 OCH1 OST6 PMT1 PMT5 STT3 SVP26*)
GO:0016050 vesicle organization	10 (*EMP24 GOS1 SEC12 SEC23 SFB3 SNC2 TLG1 UBC1 VPS4 VTI1*)
GO:0031399 regulation of protein modification process	10 (*CDC11 CLB4 DBF4 NCE102 PCL2 PTC2 PTP3 SGV1 STE7 SWE1*)
GO:0042273 ribosomal large subunit biogenesis	10 (*MAK16 REH1 RLP24 RLP7 RPL11A RPL5 RRP5 RRS1 SDA1 SQT1*)
GO:0070647 protein modification by small protein conjugation or removal	10 (*CDC16 NOT3 RAD5 RFA1 RFA2 RPL40A UBC1 UBC4 UBP16 YUH1*)
GO:0007033 vacuole organization	9 (*ATG1 ATG12 ENO1 GYP7 RDI1 TPM2 VPS21 VTI1 YHC3*)
GO:0016197 endosomal transport	9 (*ATG18 RAV1 RRT2 VPS17 VPS4 VPS55 VPS68 VPS8 YPT32*)
GO:0016570 histone modification	9 (*ACS2 ARD1 GLC7 ORC2 RPD3 RTT109 SGV1 SWD2 TAF5*)

GO:0051049 regulation of transport	9 (*AKL1 ARG81 CUP9 FPK1 GEF1 SEC12 SEC23 SLG1 TUP1*)
GO:0061025 membrane fusion	9 (*ENO1 GOS1 GYP7 PRM1 RDI1 SEY1 SNC2 TLG1 VTI1*)
GO:0070925 organelle assembly	9 (*ATG1 ATG12 DRS2 ESF2 RIA1 RPL11A RPL5 RPS31 SQT1*)
GO:0006413 translational initiation	8 (*BRF1 GCD1 GCD6 HYP2 SIS1 SUI1 SUI3 YGR054W*)
GO:0006970 response to osmotic stress	8 (*AIP1 GPD1 NHA1 RGD1 RVS161 SLG1 SSK22 TUP1*)
GO:0007031 peroxisome organization	8 (*ANT1 ATG18 BCK1 PEX14 PEX7 PEX8 SLG1 VPS34*)
GO:0007114 cell budding	8 (*BUD3 DCW1 GLC7 KCC4 TGL3 TGL4 TPM2 TWF1*)
GO:0009408 response to heat	8 (*GET3 GLC7 HSP104 NUP188 NUP84 RPD3 SLG1 YAP1*)
GO:0042594 response to starvation	8 (*AFT1 ATG1 ATG12 ATG18 NPR3 PRB1 RVS161 ZPR1*)
GO:0043144 snoRNA processing	8 (*NOP1 POP1 POP7 RPP1 RRP40 SEN1 SWD2* TRF5)
GO:0048284 organelle fusion	8 (*DYN1 GOS1 KAR2 KAR4 SEY1 SNC2 TLG1 VTI1*)
GO:0051052 regulation of DNA metabolic process	8 (*CCR4 CDC16 CHK1 RPD3 SGS1 SIR2 SPT4 SPT5*)
GO:0051604 protein maturation	8 (*ATG18 IMP2 RIM13 SPC1 SPC3 STE23 STE24 YPS1*)
GO:0006470 protein dephosphorylation	7 (*GLC7 PHO8 PPG1 PTC2 PTC5 PTP3 RTR1*)
GO:0006865 amino acid transport	7 (*AGC1 AVT7 GAP1 PUT4 SSY1 TAT2 YHC3*)
GO:0042255 ribosome assembly	7 (*DRS2 ESF2 RIA1 RPL11A RPL5 RPS31 SQT1*)
GO:0043543 protein acylation	7 *(ACS2 ARD1 MAK3 NAT3 NMT1 RTT109 TAF5*)
GO:0006869 lipid transport	6 (*DRS2 FAA1 FPK1 KES1 NPC2 PXA1*)
GO:0006887 exocytosis	6 (*ARG81 KES1 KIN1 SNC2 TPM2 YPT32*)
GO:0009451 RNA modification	6 (*DUS3 ELP4 HRR25 KTI11 NOP1 SAP190*)
GO:0048308 organelle inheritance	6 (*ARP3 MDM1 NAT3 SEY1 TPM2 VPS21*)
GO:0006352 DNA-dependent transcription, initiation	5 (*BRF1 RPT1 SIN4 SPT16 TAF5*)
GO:0006360 transcription from RNA polymerase I promoter	5 (*RPD3 SPT4 SPT5 TAP42 UTP15*)
GO:0006997 nucleus organization	5 (*DYN1 KAR2 KAR4 NUP188 NUP84*)
GO:0032200 telomere organization	5 (*RFA1 RFA2 SGS1 SUA5 SWD2*)
GO:0000902 cell morphogenesis	4 (*GLC7 KCC4 SRV2 YCK2*)
GO:0006414 translational elongation	4 (*GCN1 HYP2 RPS2 SUA5*)
GO:0006418 tRNA aminoacylation for protein translation	4 (*ISM1 KRS1 MES1 YHR020W*)
GO:0006497 protein lipidation	4 (*ATG12 MCD4 NMT1 SPT14*)
GO:0008213 protein alkylation	4 (*ORC2 SFM1 SGV1 SWD2*)
GO:0008643 carbohydrate transport	4 (*GLK1 HXT6 JEN1 STL1*)
GO:0006353 DNA-dependent transcription, termination	3 (*GLC7 SEN1 SWD2*)
GO:0006766 vitamin metabolic process	3 (*ABZ1 THI20 THI21*)
GO:0010324 membrane invagination	3 (*ATG1 ATG12 ATG18*)
GO:0032196 transposition	3 (*BRF1 RTT109 STE7*)
GO:0000054 ribosomal subunit export from nucleus	2 (*RRS1 SDA1*)
GO:0001403 invasive growth in response to glucose limitation	2 (*STE7 TPK2*)
GO:0006383 transcription from RNA polymerase III promoter	2 (*BRF1 RPC34*)
GO:0007124 pseudohyphal growth	2 (*HMS1 STE7*)
GO:0009311 oligosaccharide metabolic process	1 (*HSP104*)
