# Supplementary material for: Pichia pastoris Aft1 - a novel transcription factor, enhancing recombinant protein secretion
Source: Microb Cell Fact. 2014 Sep 3;13:120. doi: 10.1186/s12934-014-0120-5 (PMC4161868; doi:10.1186/s12934-014-0120-5)
Supplement: Additional file 4: — SDS-PAGE of carboxylesterase secreting P. pastoris strains. Clones #8-20: CBS7435 transformed with plasmid pPM2dZ30-PGAP-CE; negative control: CBS7435. [file 12934_2014_120_MOESM4_ESM.pdf]

Additional File 4. Primers for split marker (sub)fragments (Primers 1-8) for the knock out of Aft as well as the primer pair for verification of positive knock outs (Primers 9-10), which binds outside the split marker cassette on genomic DNA.

Primer		sequence	TM [°C]	sub-fragment	split marker fragment	split marker fragment size [bp]
1	A_fw	TGCATAAGAACACATCAAACACACG	68	A	AB	2424
2	A_bw	GTTGTCGACCTGCAGCGTACGTGTCAAGCAATCGGGGTG	67			
3	B_fw	CACCCCGATTGCTTGACACGTACGCTGCAGGTCGACAAC	66	B		
4	B_bw	CGGTGAGAATGGCAAAAGCTTATG	70			
5	C_fw	AAGCCCGATGCGCCAGAGTTG	68	C	CD	2360
6	C_bw	CCGTATCCTAAATGGGCAGTTGTAGTGGATCTGATATCACCTA	54			
7	D_fw	TAGGTGATATCAGATCCACTACAACTGCCCATTTAGGATACGG	67	D		
8	D_bw	GGATGAGGATGACGACGATG	66			
9	Check_fw	GCCCACCTGTTTTGAGACTGTAG	66			
10	Check_bw	TGATTTATTTTTTGTGATTTTGGACGAAG	68			
