# Supplementary material for: Pichia pastoris Aft1 - a novel transcription factor, enhancing recombinant protein secretion
Source: Microb Cell Fact. 2014 Sep 3;13:120. doi: 10.1186/s12934-014-0120-5 (PMC4161868; doi:10.1186/s12934-014-0120-5)
Supplement: Additional file 5: — Primers used for the construction of the split marker cassette for AFT1 disruption in P. pastoris. [file 12934_2014_120_MOESM5_ESM.pdf]

Primer		Sequence	TM [°C]	Sub-fragment	Split marker fragment	Split marker fragment size [bp]
1	A_fw	TGCATAAGAACACATCAAACACACG	67.9	A	AB	2424
2	A_bw	GTTGTCGACCTGCAGCGTACGTGTCAAGCAATCGGGGTG	67.03			
3	B_fw	CACCCCGATTGCTTGACACGTACGCTGCAGGTCGACAAC	66.24	B		
4	B_bw	CGGTGAGAATGGCAAAAGCTTATG	70			
5	C_fw	AAGCCCGATGCGCCAGAGTTG	68	C	CD	2360
6	C_bw	CCGTATCCTAAATGGGCAGTTGTAGTGGATCTGATATCACCTA	54			
7	D_fw	TAGGTGATATCAGATCCACTACAACTGCCCATTTAGGATACGG	66.92	D		
8	D_bw	GGATGAGGATGACGACGATG	66.01			
