# Supplementary material for: Pichia pastoris Aft1 - a novel transcription factor, enhancing recombinant protein secretion
Source: Microb Cell Fact. 2014 Sep 3;13:120. doi: 10.1186/s12934-014-0120-5 (PMC4161868; doi:10.1186/s12934-014-0120-5)
Supplement: Additional file 6: — Primers used for the verification of gene regulation in Δ aft1 and wild type. Comparison of gene regulation patterns from microarrays and qPCR for genes selected based on their regulatory behaviour. [file 12934_2014_120_MOESM6_ESM.pdf]

Additional File 6: The expression changes of some genes observed in the microarray experiment was confirmed using quantitative real time PCR.

Total RNA from the microarray experiments was subjected to DNAse digestion. Reverse transcription of mRNA and determination of transcript levels by quantitative real-time PCR were performed as described in Stadlmayr *et al.* 2010 using the primers given in **Additional Table 6A**. The gene encoding actin-related protein *ARP3* was used as reference gene for normalization. Each transcript was correlated to the *ARP3* transcript level from the same sample as an internal control. One biological replicate of the wild type strain was used as the reference strain for each relative transcript level determination using the ΔΔCt method. As can be seen in **Additional Figure 6B**, there is a high degree of correlation between microarray and qPCR results.

Additional Table 6A: Primers used for the verification of gene regulation in Δ*aft1* and wild type by qPCR**.**

**Name**	**Sequence**
AFT2_bw	AAATGTTGGATGCCTCCTTG
AFT2_for	CCACCAGAATACGTCGTCCT
FDH1_bw	GACGGTAACAACATCGGCTT
FDH1_for	GCAAATTGTTAACCACGGCT
PAS_chr4_0589_bw	CTCGACCGACAGACGGTATT
PAS_chr4_0589_for	CTTGCTCCGCTGTTAGATCC
PEX13_bw	CATCCCATAGCCACCCATAC
PEX13_for	TAAATGCTGGCCAATCTTCC
TAL1-2_bw	GAAAACTTTCGAACCCCCTC
TAL1-2_for	GAGCAGGAGCCTTTTGTGTC

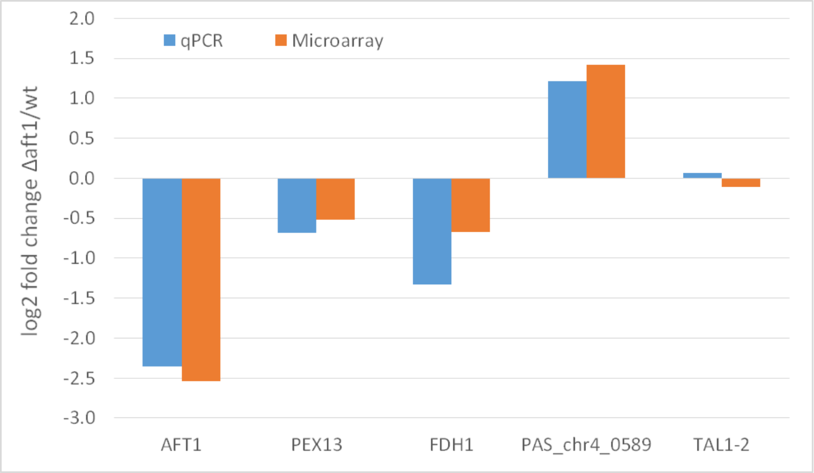

Additional Figure 6B: Comparison of gene regulation patterns from microarrays and qPCR. Genes were selected based on their regulatory behaviour. Good correlation can be seen for all analysed genes.

References:

Stadlmayr G, Mecklenbräuker A, Rothmüller M, Maurer M, Sauer M, Mattanovich D, Gasser B. 2010. Identification and characterisation of novel *Pichia pastoris* promoters for heterologous protein production. *J. Biotechnol*. 150:519–529.
